# Bimolecular Reaction Mechanism in the Amido Complex-Based Atomic Layer Deposition of HfO_2_

**DOI:** 10.1021/acs.chemmater.2c02947

**Published:** 2023-01-03

**Authors:** Giulio D’Acunto, Roman Tsyshevsky, Payam Shayesteh, Jean-Jacques Gallet, Fabrice Bournel, François Rochet, Indiana Pinsard, Rainer Timm, Ashley R. Head, Maija Kuklja, Joachim Schnadt

**Affiliations:** † Department of Physics, Division of Synchrotron Radiation Research, and NanoLund, Lund University, Box 118, 221 00 Lund, Sweden; ‡ Department of Materials Science and Engineering, University of Maryland, College Park, Maryland 20742, United States; § CNRS Laboratoire de Chimie Physique-Matière et Rayonnement, Sorbonne Université, 4 place Jussieu, 75005 Paris, France; ∥ Synchrotron SOLEIL, L’Orme des Merisiers, Saint-Aubin, BP 48, 91192 Gif-sur-Yvette Cedex, France; ⊥ Center for Functional Nanomaterials, Brookhaven National Laboratory, P.O. Box 5000, Upton, New York 11973-5000, United States; # MAX IV Laboratory, Lund University, Box 118, 221 00 Lund, Sweden

## Abstract

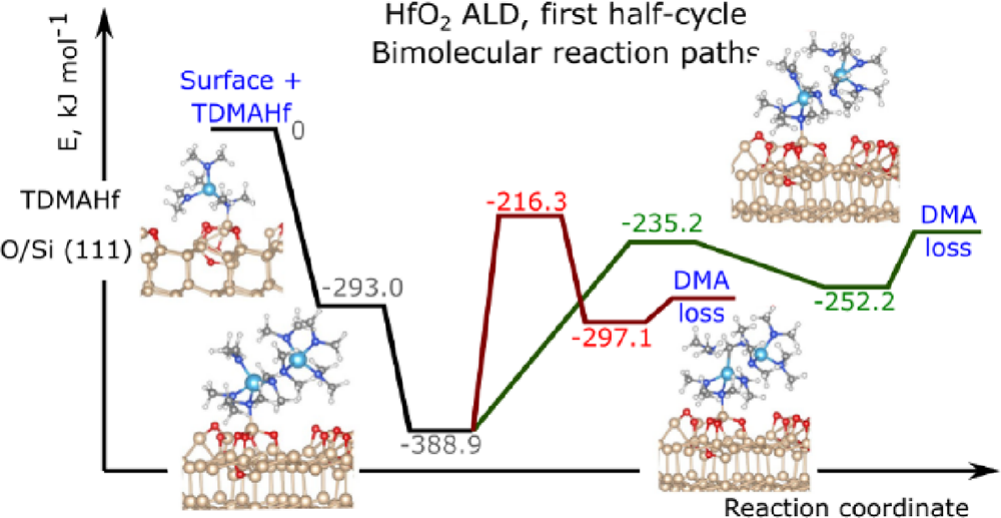

The surface chemistry of the initial growth during the first or first few precursor cycles in atomic layer deposition is decisive for how the growth proceeds later on and thus for the quality of the thin films grown. Yet, although general schemes of the surface chemistry of atomic layer deposition have been developed for many processes and precursors, in many cases, knowledge of this surface chemistry remains far from complete. For the particular case of HfO_2_ atomic layer deposition on a SiO_2_ surface from an alkylamido-hafnium precursor and water, we address this lack by carrying out an *operando* atomic layer deposition experiment during the first cycle of atomic layer deposition. Ambient-pressure X-ray photoelectron spectroscopy and density functional theory together show that the decomposition of the metal precursor on the stoichiometric SiO_2_ surface in the first half-cycle of atomic layer deposition proceeds via a bimolecular reaction mechanism. The reaction leads to the formation of Hf-bonded methyl methylene imine and free dimethylamine. In addition, ligand exchange takes place involving the surface hydroxyls adsorbed at defect sites of the SiO_2_ surface.

## Introduction

Atomic layer deposition (ALD) is a thin-film growth technique that is essential to the further miniaturization of semiconductor devices due to its ability to produce ultrathin transition metal oxide films in a highly controlled fashion on both flat and structured surfaces.^1^ In ideal binary metal oxide ALD, the controllability of the growth process derives from the self-limiting nature of the adsorption and surface reaction of two gas-phase precursors—a metal and an oxygen source—that are alternately introduced to the target surface.^2^ The self-limiting nature of the precursor/surface interaction is thought to be enabled by a typically straightforward chemical reaction scheme, based on a ligand exchange reaction mechanism. The scheme can be derived from the known solution chemistry of the precursors.^3^

In reality, however, the fact that the precursors are provided to the surface in the gas rather than the liquid phase and the involvement of the surface in the chemical reaction imply that the chemistry of the growth process is considerably more complex than what the proposed reaction schemes assume, and knowledge about the exact surface chemical processes is very limited.^4−6^ In consequence, it is difficult to control undesirable side reactions and to improve the quality of the grown films in a deliberate fashion. Conventional surface science methods provide an avenue toward an improved understanding, but they are typically hampered by being limited to high-vacuum environments. The application of *operando* methods provides entirely new possibilities for developing a deeper understanding of the ALD surface chemical process. These methods were developed primarily within the catalysis research domain and refer to the spectroscopic characterization of a working catalyst under realistic operating conditions in general and realistic pressure conditions in particular. Catalytic *operando* measurements require also the simultaneous acquisition of reaction data.^7^

Since the term *in situ* experiment is typically used in the ALD community to denote an experiment in which the characterization of sample growth is performed in between cycles and often in vacuum but in the same instrument in which ALD is carried out and thus without exposure of the sample to air, we employ the term *operando* to clearly distinguish the time-resolved characterization during growth and at realistic or close to realistic pressure conditions from such *in situ* characterization without time resolution, e.g., by infrared spectroscopy,^8^ ellipsometry,^9^ X-ray fluorescence, X-ray scattering,^10^ and X-ray photoelectron spectroscopy.^11^ It needs to be noted, however, that the term *in situ* is sometimes used in the ALD community to even describe experiments in which the characterization of the sample is performed during the deposition, often without but sometimes also with time resolution. For example, the time-resolved characterization during deposition by quartz crystal microbalance measurements,^12^ quadrupole mass spectrometry,^13^ pyroelectric calorimetry,^14^ and ellipsometry^15^ has been termed *in situ*. *In situ* methods, whether performed during the deposition or in between the half-cycles and whether carried out in a time-resolved manner or not, have been providing highly valuable information for a long time. Only recently, these techniques have been joined by spectroscopic *operando* techniques (infrared spectroscopy^16^ and APXPS^17−19^) that deliver direct chemically specific information and that can be used to follow the surface chemistry of the ALD process in real time and at processing pressures equal or similar to those in an ALD reactor.

Here, we report the application of ambient-pressure X-ray photoelectron spectroscopy (APXPS) (see, e.g., refs (20, 21)) to the study of the first ALD cycle of HfO_2_ on an oxidized Si(111) surface from tetrakis(dimethylamido)hafnium [TDMAHf, Hf(N(CH_3_)_2_)_4_] and water. The *in situ* oxidized Si(111) surface was chosen instead of a native SiO_2_ surface since it can be prepared in a much more controlled and very clean way, at the same time as its surface structure is very similar to that of both α-Si(001) and of amorphous SiO_2_ (cf. Figure S2 and refs (22, 23)). Hence, the top layer of the oxidized Si(111) surface can be regarded as a single-layer oxide, which, in similarity to metal-supported two-dimensional silicon oxide, is highly regular. SiO_2_ hydroxylates easily at, and only at, defect sites, but stoichiometric SiO_2_ remains free from hydroxyls upon exposure to water.^24^ Therefore, the use of a thin SiO_2_ model film allows us to monitor the ALD chemistry at the regular lattice sites of SiO_2_.

The ambient-pressure X-ray photoelectron (APXP) spectra of all relevant core levels were recorded in a cyclic fashion, where a single sequence of spectra was acquired within 13 s. In other words, a 0.08 Hz measurement rate was achieved, which so far is unprecedented in the *operando* monitoring of ALD and which allows us to follow the evolution of the surface chemical species, both in terms of the state of the Hf ions, the ligands, and their reaction products. We can establish the order of appearance of the different species and follow their temporal changes, and we can truly correlate the state of the Hf ions with the states of the precursor ligands. The surface chemical species are identified on the basis of their spectral fingerprints. Calculations of TDMAHf adsorption and decomposition were carried out using methods of density functional theory (DFT) to aid the interpretation of the experimental results.

The HfO_2_/SiO_2_ system is investigated due to its relevance to the interface of an ultrathin high-κ HfO_2_ film with Si, where the Si surface is oxidized deliberately to avoid the potential formation of an intermixed interfacial layer as a result of oxygen diffusion in any high temperature treatment following gate dielectric fabrication.^25^ HfO_2_ can be grown by ALD using different metal precursors (cf. ref (2). TDMAHf is one of the standard precursors, not least because its handling is comparatively easy in comparison to that of other metal precursors.^26^ In ALD, it is used at pressures in the range from around 10^–3^ to 1.3 mbar.^27^ Here, we used a TDMAHf pressure of 0.02 to 0.03 mbar, within the range of reported operating pressures of TDMAHf-based ALD processes. In principle, APXPS experiments could have been performed at more typical pressures in the 0.1 to 1 mbar range; keeping to the lower end of the reported operating pressure range has the advantage, however, that the surface chemical processes are slower and more easily followed in detail [cf. Methods and Section S2 of the Supporting Information (SI)].

We find from our *operando* APXPS measurements that decomposition pathways beyond amido ligand removal in a ligand exchange scheme play an important role in the initial ALD of HfO_2_ from TDMAHf and water. In the first TDMAHf half-cycle, a large fraction of the amido ligands are converted into Hf-bonded imines; their formation is TDMAHf coverage-dependent and occurs first when the coverage is sufficiently high. This behavior provides strong evidence of the bimolecular nature of the reaction mechanism. DFT fully supports the notion of TDMAHf dimer formation on SiO_2_ surfaces followed by the decomposition of TDMAHf in the dimer in a bimolecular reaction mechanism. The bimolecular decomposition pathway is significantly more favorable energetically than any unimolecular reaction.

## Methods

### Sample Preparation

A highly phosphorus-doped Si(111) sample was degassed in ultrahigh vacuum for 11 h at around 600 °C. The temperature was controlled by a thermocouple placed in good electrical contact with the sample. Then, the native oxide was removed by flash annealing to 1050 °C. Finally, the room-temperature sample was exposed to 45 L of O_2_ gas (1 L = 1 Langmuir = 1.33 × 10^–6^ mbar s) to obtain an oxidized Si(111) surface.

### Precursor Delivery

Pure TDMAHf (99.99%, Strem Chemicals) and ultrapure water were used as precursors for the ALD experiment. The two precursors were dosed via a custom-made gas delivery system [cf. Figure S1 in the SI] heated at 60 °C, through two separate gas lines. The gas lines were equipped with Swagelok diaphragm valves with nominal opening and closing times on the order of 5 ms. Before each half-cycle, the corresponding precursor line was pumped out.

### APXPS

APXPS was conducted at the NAP-XPS end station on the TEMPO beamline at the SOLEIL synchrotron, France. The end station is equipped with a SPECS Phoibos 150 NAP electron energy analyzer. The first differential pumping stage of the analyzer was separated from the sample environment by a nozzle with a diameter of 0.3 mm. During exposure of the sample (at 280 °C) to TDMAHf and water vapor, the Si 2p, C 1s, N 1s, Hf 4f, and O 1s core levels were recorded at a fixed photon energy of 700 eV. The snapshot mode of the electron energy analyzer was used with a fixed pass energy of 50 eV, which implied a kinetic energy window of around 9 eV, and a total set of core levels was measured within 13 s. The binding energy scale of all spectra was calibrated to the Si 2p_3/2_ bulk peak at 99.3 eV (ref (31)), in good agreement with literature values (refs (32−36)). The overall experimental resolution was about 200 meV. The pressure during the experiment was monitored by a gauge mounted on the ALD chamber. For the first half-cycle, two metal precursor (TDMAHf) pulses of around 15 s time length were executed, leading to a pressure of 0.02 mbar. The pressure was retained after the pulses, resulting in a longer exposure of the sample to TDMAHf for around 15 min. During this time, the metal precursor coverage increased to around three monolayers (see analysis below). During the most relevant initial 3 min of exposure, the molecular coverage went up to 0.35 (cf. analysis below and Figure 2c). In terms of the number of TDMAHf molecules impinging on the surface, this 3 min exposure at 0.02 mbar corresponds to a 6 s exposure under standard ALD experiments with a typical TDMAHf pressure of 0.6 mbar (cf. Section S2 in the SI).

**Figure 1:**
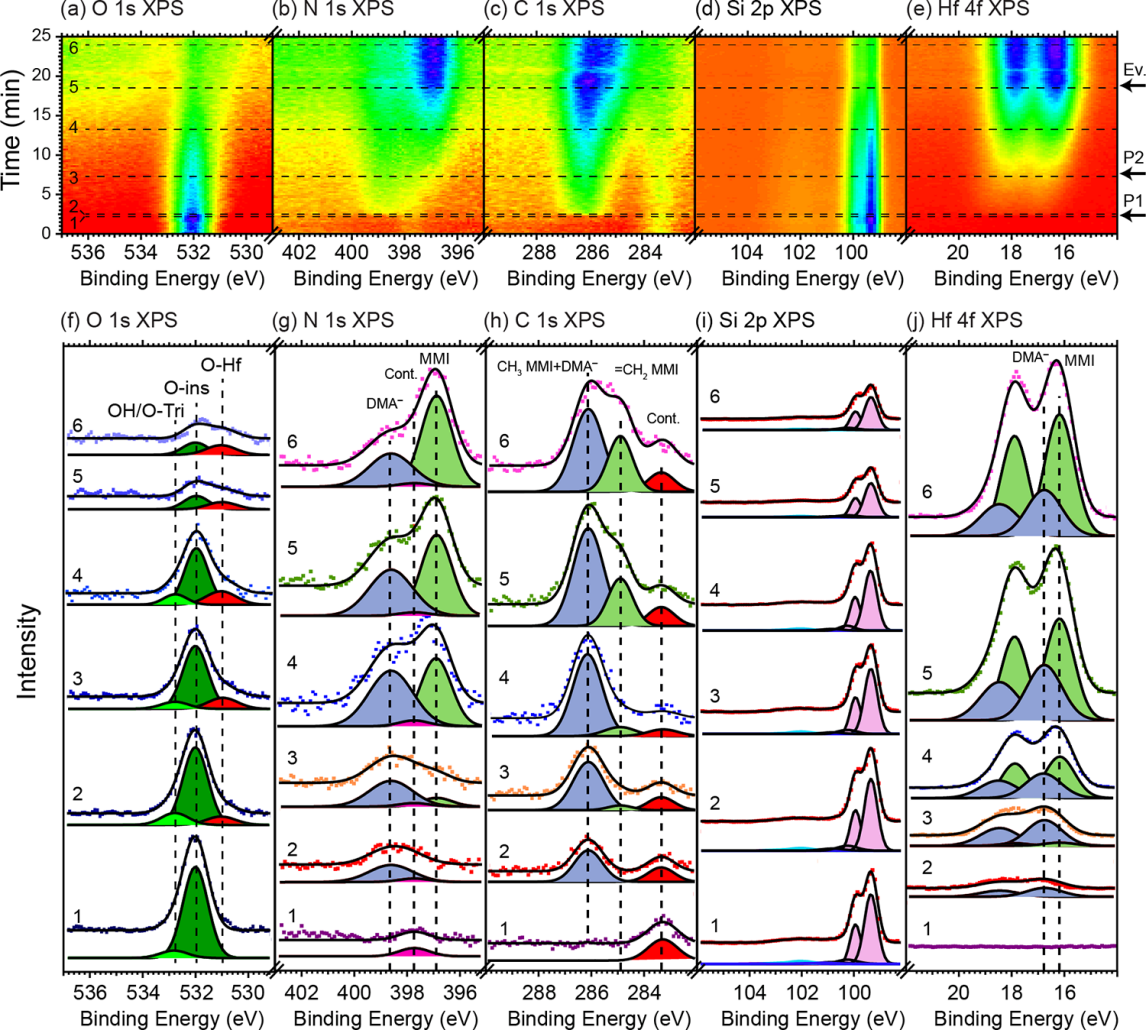
Series of O 1s, N 1s, C 1s, Si 2p, and Hf 4f APXP spectra measured during the first ALD (metal) half-cycle. The different core levels were measured in sequence. (a–e) Image plots of the indicated core levels. The intensity is represented by a false color scale, where violet represents maximum and red minimum intensity. The times of TDMAHf dosing (pulses P1 and P2) and pump-out (Ev.) are indicated. (f–j) Selected spectra as indicated by the numbered dashed lines in (a–e) refer to (1) pristine sample, (2) after the first TDMAHf pulse resulting in a pressure of 0.02 mbar, (3) before the second TDMAHf pulse, (4) after the second TDMAHf pulse, (5) before the evacuation, and (6) after the evacuation.

For the second half-cycle, water was dosed three times (60, 20, and 20 s) at a pressure of around 0.35 mbar.

The data analysis was conducted entirely in Igor Pro (more details in SI, Section S4).

### DFT

Solid-state periodic calculations were performed by employing DFT with the optPBE-vdWS15-S19 functional, which includes corrections for van der Waals interactions, as implemented in the VASP code.^37−39^ The projector-augmented wave (PAW) method^40^ was used. Solid-state calculations were carried out on the oxidized Si(111)-(7 × 7) and a SiO_2_(001) surface (Figure S2). For more details, please refer to SI, Section S3.

## Results

### First ALD Half-Cycle: Exposure to TDMAHf

All APXP spectra obtained during the TDMAHf half-cycle can be found in the form of image plots in the top panels (a–e) of Figure 1; panels (f–j) show selected spectra together with least-squares curve fits from a global curve fitting procedure for each of the core levels in (a–e). Initially, two components are discerned in the O 1s spectrum. The main component at 532.0 eV binding energy is due to the so-called “O-ins” oxygen species of the oxidized Si(111) surface, which are oxygen atoms inserted into the back bonds of surface Si adatoms.^41^ The component at 532.8 eV can have two different origins: it can be due to the “O-tri” oxygen surface species of the oxidized Si(111) surface, i.e., due to oxygen atoms that bond between the first- and second-layer Si atoms,^41^ or it can be due to surface hydroxyls on the oxidized film (cf. Section S5e in the SI). The initial N 1s and C 1s spectra exhibit weak components at 397.7 and 283.3 eV, respectively; they stem from residual gas adsorption as a result of preceding experiments in the same experimental setup. The binding energies of peak components are in agreement with Si–N and Si–C chemical environments.^42,43^

At *t* ≈ 2.5 min, a TDMAHf pulse (0.02 mbar) is delivered to the surface. The pressure is retained after the pulse. At *t* ≈ 8 min, a second pulse is provided, which leads to a pressure increase to 0.03 mbar. This pressure is maintained until evacuation of the chamber at *t* ≈ 18 min. The second pulse is provided to ensure that enough material is available for adsorption saturation but is found not to contribute any further to the evolution of the surface species.

The delivery of TDMAHf to the surface at *t* ≈ 2.5 min leads to the immediate appearance of new components in all spectral regions except the Si 2p one. The binding energies of the new components are 398.6 eV in the N 1s, 286.1 eV in the C 1s, 16.8 eV in the Hf 4f_7/2_, and 531.0 eV in the O 1s spectra. The N 1s and C 1s peaks can be identified with the nitrogen atoms and methyl groups of the dimethylamido (DMA^–^) ligands.^44^ The C 1s:N 1s intensity ratio is 1.5, close to the expected value of 1.76 derived in Section S6 of the SI. In the Hf 4f spectral region, a single peak is observed; based on the observation of DMA^–^ in the N 1s and C 1s spectra and since a complete initial decomposition of the TDAMHf complex is unlikely, the single Hf 4f component is assigned to surface Hf ions bonded to DMA^–^ ligands.

**Figure 2:**
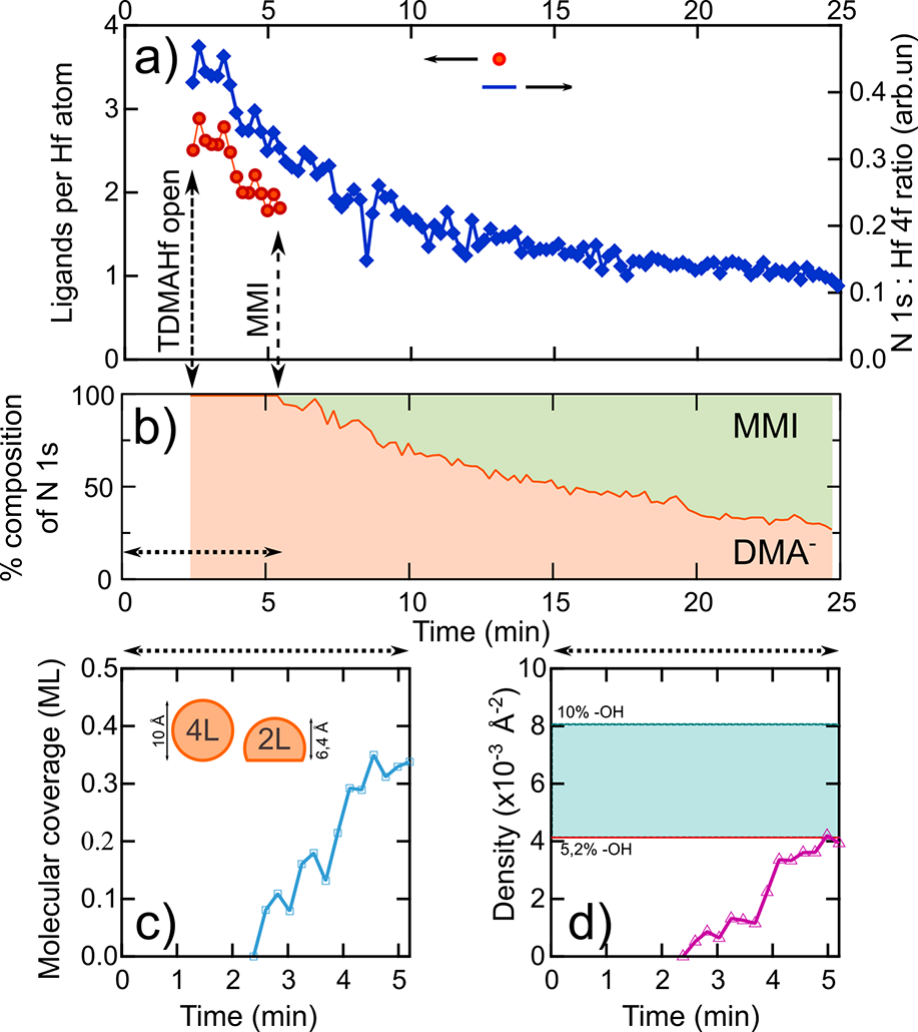
(a) Blue curve: N 1s:Hf 4f intensity ratio derived from the spectra in Figure 1. Red markers: number of ligands retained per Hf ion on the surface. (b) Fraction of MMI (green) and DMA^–^ (orange) surface species, derived from the components in the N 1s core level. (c) Molecular coverage of the SiO_2_ surface during the early stage of the first half-cycle. The inset depicts the molecular model that underlies the coverage calculation. In this model, we assume the presence of both fully intact surface-adsorbed TDMAHf complexes (“4 L”, L = DMA^–^) and of partially dissociated O_2_-Hf(DMA^–^)_2_ surfaces complexes (“2 L”). Details are provided in SI, Section S5c. (d) Density of partially dissociated O_2_-Hf(DMA^–^)_2_ (“2 L”) complexes adsorbed on the SiO_2_ surface during the early stage of deposition. The dashed blue line represents the maximum possible surface hydroxyl density on the SiO_2_ surface, derived from the O 1s spectrum prior to TDMAHf deposition (see Section S5e in the SI). The red line corresponds to the maximum density of the O_2_-Hf(DMA^–^)_2_ surface complexes.

The N 1s:Hf 4f intensity ratio after the first TDMAHf pulse can be evaluated to determine the average number of DMA^–^ ligands retained per Hf ion at the surface (cf. SI, Section S5c). The result of the evaluation is shown in Figure 2a. The figure shows that, on average, 2.5 DMA^–^ ligands are retained per surface-adsorbed hafnium ion after the first TDMAHf pulse. Hence, a majority of the vapor TDMAHf complexes dissociate partially upon adsorption of the surface to form −Hf(DMA^–^)_*x*_ surface complexes (*x* < 4). At the same time, a sizeable minority of surface-adsorbed TDMAHf complexes remain fully intact (SI, Section S5c). The occurrence of dissociated −Hf(DMA^–^)_*x*_ surface complexes as derived from the N 1s and Hf 4f APXPS signals is in good agreement with the occurrence of a new O 1s component at around 531 eV binding, attributable to the oxygen-bonded surface complexes, O_*y*_–Hf(DMA^–^)_*x*_ (*x* < 4). It is noteworthy that the relatively low binding energy of the component is that expected for a HfO_2_ type of environment of the hafnium ions rather than a (low Hf content) hafnium silicate.^44,45^ In contrast, the Hf 4f binding energy of 16.8 eV is around 1 eV lower than that expected for a HfO_2_ type of environment, which is in line with what we have observed for the initial interaction of TDMAHf with the native oxide on InAs.^16,46^ We conclude that it is coincidental that the O 1s binding energy of the O_*y*_–Hf(DMA^–^)_*x*_ surface complexes is virtually the same as that of HfO_2_ and not a sign of HfO_2_ formation. Similarly, the presence of both fully intact TDMAHf and partially dissociated TDMAHf surface complexes, where the latter forms one or more bonds to surface oxygen atoms, would be expected to lead to the appearance of at least two different components in the Hf 4f spectra. Only one component is observed, and we suggest the similarity of the different Hf 4f binding energies to be coincidental. We note that the attribution to DMA^–^-bonded Hf ions is in line with the TDMAHf gas-phase spectra in Section S5a of the SI for which the N 1s–Hf 4f binding energy difference is approximately 382.0 eV, while it is 381.8 eV for the surface-adsorbed TDMAHf complexes. The small deviation is likely a sign that the main fraction of the surface-adsorbed complexes is partially dissociated as outlined above.

Around 3 min after the first TDMAHf pulse, i.e., at *t* ≈ 6 min, components related to a new chemical species are seen to gain intensity in the spectra in Figure 1. This new species develops clearly after the start of the exposure of the SiO_2_ sample to TDMAHf but also clearly before the start of the second TDMAHf pulse. Obviously, in the present experiment, it is observed during the very first TDMAHf pulse as a result of prolonged exposure times in comparison to the standard pulsed ALD operation mode. Under the latter conditions, the species would require a larger number of pulses to develop. The binding energies of the new components are 396.9 eV in the N 1s, 284.9 eV in the C 1s, and 16.2 eV in the Hf 4f_7/2_ spectra. The N 1s and C 1s spectra can be used to identify the new surface species as methyl methylene imine (MMI).^18,19,44^ Accordingly, also, the new Hf 4f component is assigned to Hf(MMI)(DMA^–^)_*z*_ surface complexes (*z* = 0, 1, or 2, where *z* = 2 corresponds to the reaction of a fully intact TDMAHf complex and *z* = 0 and 1 to the reaction of a O_*y*_–Hf(DMA^–^)_*x*_ surface complex, cf. the DFT results). Eventually, MMI becomes the dominating surface species, as is seen from Figure 2b.

MMI is formed in an insertion reaction in which the β-hydride on one of the ligands of TDMAHf is transferred to one of the other ligands to form dimethylamine (N(CH_3_)_2_H, DMA).^19,44,47,48^ DMA is expected to be observed in the N 1s spectra at a binding energy that is higher than that of DMA^–^;^19,44,49^ no such component is seen in the spectra of Figure 1. The absence of any surface-adsorbed DMA is, however, explained straightforwardly: the relatively high sample temperature, 280 °C, is more than 100 °C higher than the expected desorption temperature of DMA.^46^

**Figure 3:**
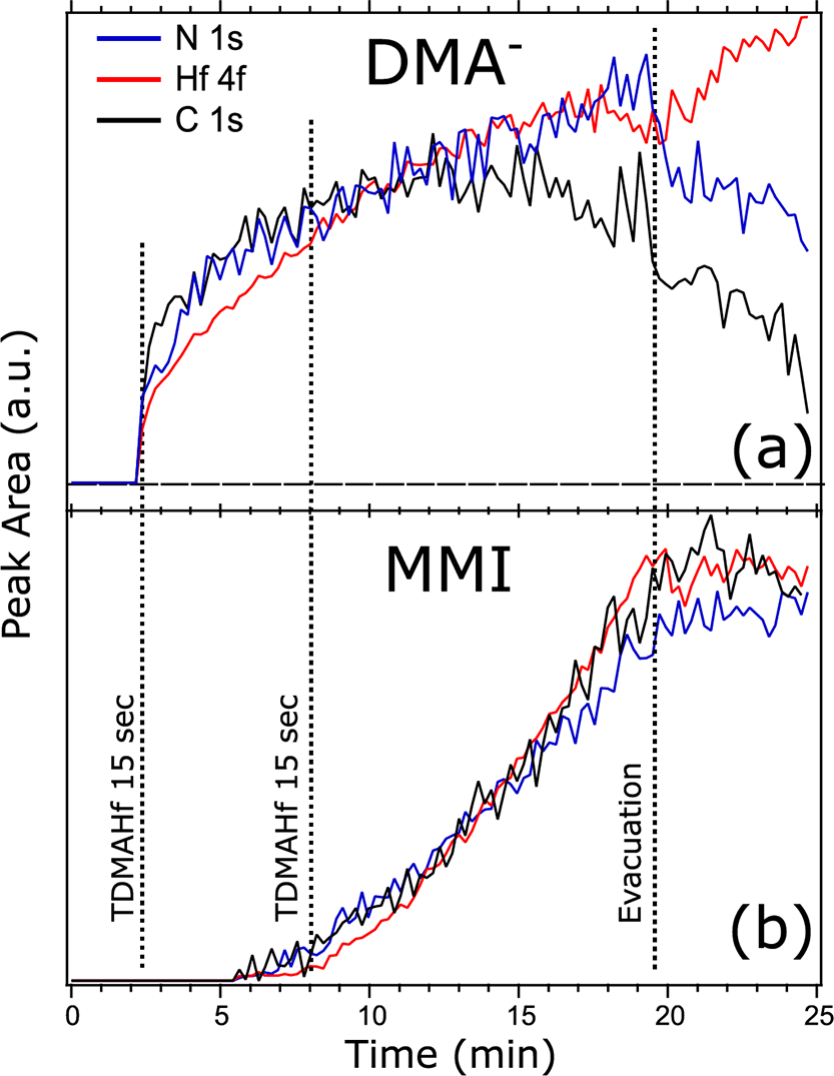
Evolution of the intensities of the Hf 4f, N 1s, and C 1s components during the initial metal half-cycle. The times of the TDMAHf pulses and pump-down of the chamber are indicated. Note that the pulses increase the pressure to a constant value, 0.02 mbar after the first TDMAHf pulse and 0.03 mbar after the second. (a) Evolution of the intensities of the N 1s, C 1s, and Hf 4f components related to DMA^–^. (b) Evolution of the intensities of the N 1s, C 1s, and Hf 4f components related to MMI.

The N 1s, C 1s, and Hf 4f lines can be deconvoluted according to the MMI and DMA^–^ contributions to the core-level intensities. Figure 3 summarizes the evolution of the components of the two different surface species. Clearly visible is that the deconvolution yields a valid result, at least during the first 15 to 20 min of TDMAHf exposure: the intensities of the N 1s, C 1s, and Hf 4f components due to the MMI species follow the same trend and so do the intensities of the components due to DMA^–^. Furthermore, it is seen that the DMA^–^ intensities develop most strongly during the first 5 min of TDMAHf deposition and then level off, while MMI formation sets in first after around 5 min of measurement time and continues until evacuation. The development is mirrored by the increasing dominance of the MMI surface species toward the end of the measurement time (Figure 2b).

**Figure 4:**
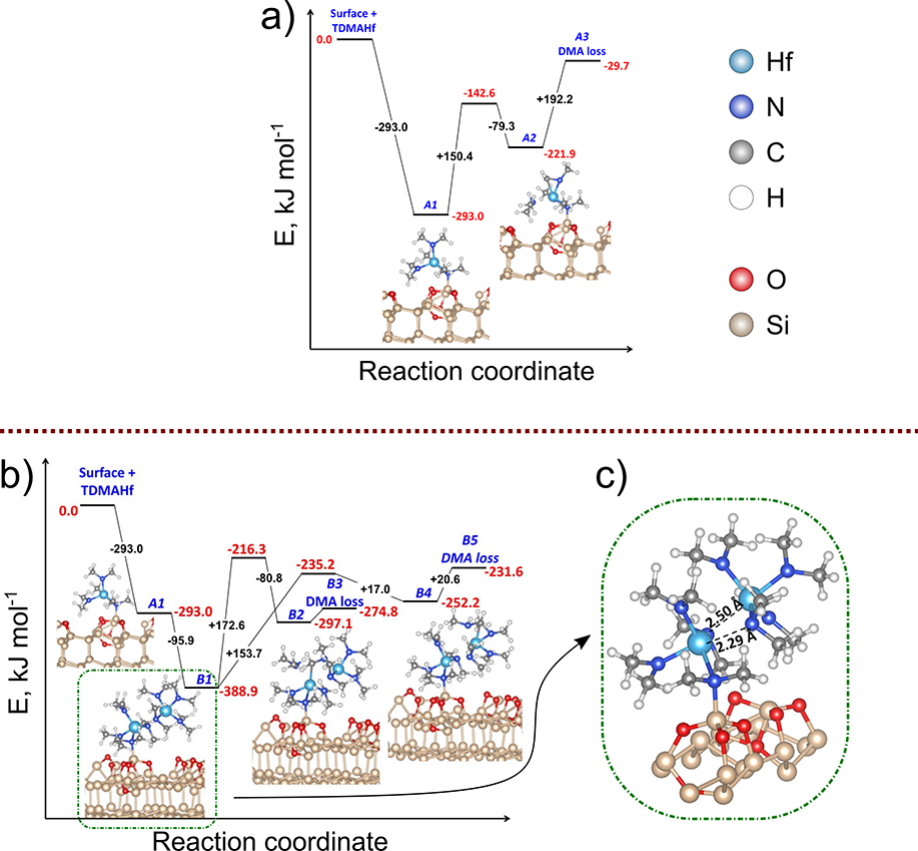
(a) Decomposition of TDMAHf via elimination of the DMA molecule. (b) Decomposition of a TDMAHf dimer on a Si(111)-(7 × 7) surface via elimination of the DMA molecule. (c) Structure of the TDMAHf dimer (configuration B1).

DFT provides further insight into the ALD surface reaction mechanism during the first metal half-cycle. Previous mechanistic studies of the interaction of alkylamido metal complexes with surfaces have focused on surfaces that either are highly reactive, such as the Si(100) surface (see, e.g., ref (50)), or that readily provide hydrogen atoms to a surface chemical reaction, such as hydrogen-, amine-, or hydroxyl-terminated Si surfaces (see, e.g., refs (48, 51, 52)). To our knowledge, no previous DFT study has been concerned with the interaction of alkylamido metal complex precursors with relatively inert surfaces such as a regular silicon oxide surface, which, as pointed out above, is not hydroxylated even upon exposure to water. In order to match the experimental conditions, we used a regular oxidized Si(111)-(7 × 7) surface as our model system (see SI, Section S3, for details). Due to its size, TDMAHf physisorbs on this surface with a rather high adsorption energy, 293.0 kJ/mol (Figure 4). No ligand exchange-type reaction pathway exists for the reaction of the surface-adsorbed TDMAHf complexes since no suitable reaction partner is available on the surface. The only viable, and overall exothermic, unimolecular reaction pathway is via the intramolecular mechanism depicted in Figure 4a; it leads to the formation of one MMI ligand and surface-adsorbed DMA, which desorbs from the surface in the last reaction step. At an overall barrier between the intact, surface-adsorbed TDMAHf complex and the final reaction products of 263.3 kJ/mol, the reaction is, however, not particularly favorable. The energetics of the unimolecular surface reaction mirror the unfavorable energetics of the unimolecular gas-phase decomposition, which is endothermic and requires 268.2 kJ/mol for the pathway via MMI formation and removal of DMA (SI, Section S6a).

A considerably more favorable decomposition pathway of TDMAHf adsorbed on the oxidized Si(111) surface implies formation of a TDMAHf dimer and a subsequent bimolecular decomposition reaction. The formation of TDMAHf dimers is viable even in the gas phase,^45^ and it is observed in the solid state,^53^ but, to our knowledge, the further decomposition has not been studied previously. The formation of the TDMAHf dimer on the oxidized Si(111) surface is a favorable process (Figure 4b, configurations A1–B1) at a calculated formation energy of −95.9 kJ/mol. In the dimer, we find an interaction between the Hf ion of one of the TDMAHf complexes and a nitrogen atom of one of the DMA^–^ ligands on the other complex (Figure 4c). For the subsequent decomposition of the dimer, resulting in a Hf-bonded MMI ligand and DMA, we have identified two plausible mechanisms. The first mechanism (Figure 4b, configurations B1–B3) involves as the first reaction step an intermolecular hydrogen transfer from a DMA^–^ ligand of one TDMAHf molecule to a DMA^–^ ligand of the other TDMAHf molecule in the dimer. The reaction step is endothermic at 91.8 kJ/mol and activated with a barrier of 172.6 kJ/mol. Subsequent removal of the formed DMA molecule into the gas phase requires another 22.3 kJ/mol. The second mechanism (Figure 4b, configurations B1–B4–B5) involves intramolecular hydrogen transfer between two DMA^–^ ligands of the same TDMAHf molecule to form an MMI ligand and DMA. The activation barrier of this reaction step is 153.7 kJ/mol, and the intermediate B4 configuration lies 136.7 kJ/mol above the initial state B1. Subsequent removal of DMA requires 20.6 kJ/mol.

Hence, the calculations suggest that the decomposition of TDMAHf on the oxidized Si(111) surface may proceed via uni- or bimolecular mechanisms, with the bimolecular mechanisms being energetically more favorable by a significant amount. All reaction mechanisms entail formation of MMI and DMA. The formed DMA desorbs into the gas phase. An evaluation of the reaction kinetics based on first- and second-order models for the uni- and bimolecular reactions, respectively, shows that the conversion rate at the experiment temperature of 280 °C is sufficiently high to explain the experimental observation of MMI formation through the bimolecular reaction mechanisms on the timescale of the experiment. The model, which is detailed in SI, Section S8, thus shows that the relatively high reaction barriers predicted by the calculations can be overcome at the experimental temperature.

DFT obtains the same results also for decomposition of TDMAHf on a stoichiometric SiO_2_(001) surface (SI, Section S7d): the reaction mechanism is the same as in the gas phase and on the oxidized Si(111) surface. Again, the bimolecular elimination of DMA from a TDMAHf dimer requires a considerably lower energy (130–160 kJ/mol) than a unimolecular elimination from a single TDMAHf complex (260–270 kJ/mol). *A posteriori*, this result also justifies the use of the oxidized Si(111) surface as a proper model for a stoichiometric SiO_2_ surface.

### Second Half-Cycle: Exposure to H_2_O

**Figure 5:**
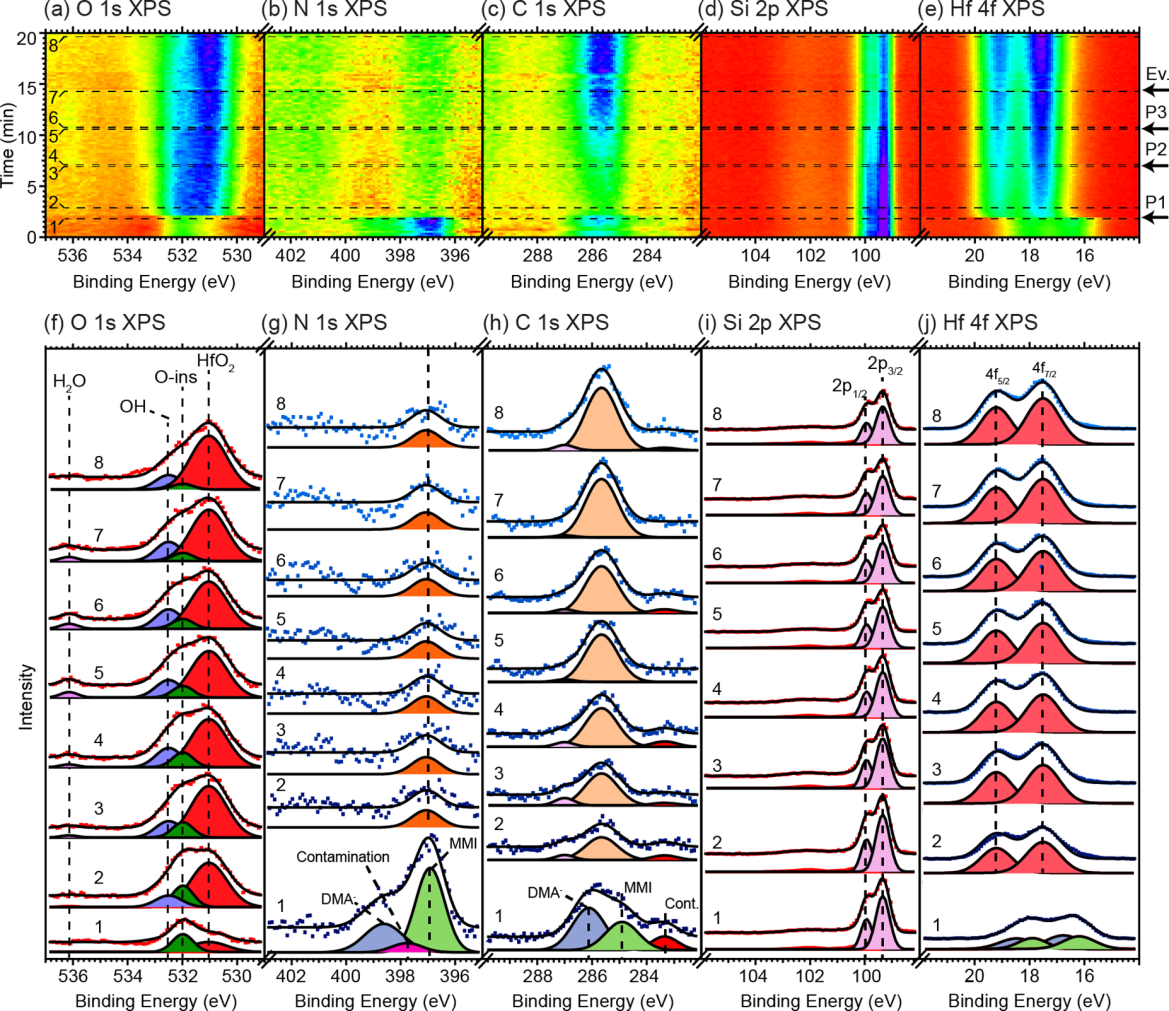
Series of O 1s, N 1s, C 1s, Si 2p, and Hf 4f APXP spectra measured during the second ALD (water) half-cycle, measured in sequence as during the metal half-cycle. The same color scale as in Figure 1 applies. (a–e) Image plots of the indicated core levels. The times of water dosing (pulses P1, P2, and P3) and pump-out (Ev.) are indicated by arrows. (f–j) Selected spectra are indicated by the numbered lines in (a–e). Spectra 1 and 2 were recorded before and after the first water pulse, spectra 3 and 4 before and after the second water pulse, spectra 5 and 6 before and after the third water pulse, and spectra 7 and 8 before and after evacuation. The component at 536.1 eV binding energy in the O 1s spectra is the water vapor line.^54^ All other lines are due to surface species.

The APXP spectra in Figure 5 were recorded during the second ALD half-cycle, i.e., during the exposure of the surface to water following the TDMAHf half-cycle and evacuation of the chamber to a pressure in the low 10^–6^ mbar regime. The water was pulsed three times to ensure that all the reactions took place; however, it appears that only the first one led to changes on the surface. Please note that a new time axis is chosen in Figure 5, separate from that of the TDMAHf half-cycle. Exposure to the first water pulse at *t ≈* 2.5 min, leading to a H_2_O pressure in the chamber of ∼0.35 mbar, induces an immediate change of the Hf 4f_7/2_ binding energy to 17.5 eV, which is in exact agreement with literature values for HfO_2_.^28^ The N 1s and C 1s signals are almost suppressed, which provides evidence for an almost complete elimination of the ligands—both DMA^–^ and MMI—from the surface. Residual intensity is, however, found in both the C 1s and N 1s spectra, with binding energies that are different from those of the DMA^–^ and MMI signals (the new components have binding energies of 285.7 and 287.0 eV in the C 1s spectra and 397.0 eV in the N 1s spectra). The O 1s line also changes considerably: the peak related to Hf-bonded oxygen at 531.0 eV grows strongly in intensity and can now be assigned to HfO_2_. In addition, we attribute a new component at a binding energy of slightly more than 532.5 eV to surface hydroxyls.

The Hf 4f signal is, however, seen to continue to increase in intensity, and also, the C 1s signal (and to a lesser degree the N 1s signal) is observed to regain intensity immediately after the water pulse. These spectral intensity increases are a sign of adsorption of residual vapor-phase TDMAHf on the surface, which reacts directly with the water. This undesired reaction is likely favored by the fact that the experiment was not performed in a dedicated ALD reactor and a typical ALD scheme with short precursor pulses but in a large chamber and with long precursor exposure times. During the water half-cycle, TDMAHf was certainly available at a partial pressure of around 10^–6^ mbar; according to basic gas kinetics, such a partial pressure is sufficient to cover the sample surface with TDAMHf adsorbates within around 1s.

## Discussion

The APXPS data presented above show clearly that MMI is formed during the first half-cycle of the initial ALD of HfO_2_ on an oxidized Si(111) surface from TDMAHf and water. The observation that MMI is not formed directly after exposure of the oxide surface to TDMAHf but rather after an incubation period during which an increasing amount of Hf-(DMA^–^)_*x*_ (*x* ≤ 4) surface complexes is formed is highly relevant and one of our key findings: transfer of the β-hydride of one of the DMA^–^ ligands to another one, which leads to the formation of the MMI and DMA products, proceeds first when a sufficiently high coverage of Hf complexes is reached on the SiO_2_ surface. This finding in itself provides strong evidence that the chemical reaction toward MMI requires two TDMAHf reaction partners, i.e., that the reaction mechanism is of a bimolecular nature. It has been observed previously that metal precursor dimer formation may take place^55−57^ and that it may influence the ALD surface chemical reaction;^55,56^ the previously observed types of influences are, however, steric in nature, while we here, for the first time, observe that dimer formation is a prerequisite for the ALD surface chemical reaction to take place in a bimolecular reaction mechanism.

The DFT results support the notion that the reaction pathway for TDMAHf complexes after adsorption on the stoichiometric oxidized Si(111)-(7 × 7) surface (and likewise on a stoichiometric SiO_2_ surface) is via the transfer of a β-hydride to another DMA^–^ ligand. This reaction is considerably more favorable in a dimer-based bimolecular reaction than a unimolecular reaction. For the stoichiometric, and thus nonhydroxylated, SiO_2_ surface, APXPS and DFT together establish the bimolecular transfer of a β-hydride to another DMA^–^ ligand as the mechanism for TDMAHf decomposition. This bimolecular reaction mechanism should be possible on most inert surfaces in general and on nonhydroxylated surfaces in particular.

The molecular surface coverage required for the bimolecular decomposition reaction to start is, however, surprisingly high: Figure 2c shows that the reaction sets in first when a coverage of 35% is reached (see SI, Section S5c, for how the coverage was derived). Statistically, a much lower coverage should be sufficient to initiate the formation of dimers of the Hf-(DMA^–^)_*x*_ (*x* ≤ 4) surface complexes and the bimolecular decomposition of these dimers. The key to understanding this surprising finding is the realization that another competing decomposition reaction takes place on the surface. The evaluation of the N 1s:Hf 4f intensity ratio [blue curve in Figure 2a] in terms of the number of retained ligands per surface-adsorbed Hf ion [red markers in Figure 2a] shows clearly that a decomposition reaction, different from the coverage-dependent bimolecular insertion reaction, sets in already at the very beginning of TDMAHf adsorption. The results of the DFT calculations make it clear that no alternative reaction pathway is available on the stoichiometric SiO_2_ surface. Hence, the conclusion must be that this second decomposition reaction does not take place on stoichiometric portions of the surface but instead at defect sites. Indeed, defect sites of the SiO_2_ surface may accommodate surface hydroxyls, which would be readily available for ligand exchange reactions with the Hf complexes that adsorb on the surface.

As already outlined above, surface hydroxyls are not easily identified in the available APXP spectra since their O 1s signature, at a high binding energy, overlaps with that of the O_tri_ species of the support. If we assume that a fraction, or all, of the high-binding energy component in the O 1s spectrum of the clean crystal (prior to TDMAHf deposition) is due to surface hydroxyls rather than O_tri_ species, then we can provide an upper limit for the surface hydroxyl density. The details of this procedure are provided in Section S5e of the SI, and the results are shown in Figure 2d: the maximum surface hydroxyl density that is compatible with the appearance of the O 1s spectrum before TDMAHf deposition is approximately 8 × 10^–3^ Å^–2^.

Concentrating on the first couple of minutes of interactions of the SiO_2_ surface with TDMAHf until the formation of MMI sets in, we find that the surface coverage of partially dissociated complexes, which have reacted with surface hydroxyls to form O_*y*_-Hf(DMA^–^)_*x*_ surface complexes, increases to approximately 5 × 10^–3^ Å^–2^ during this time (Figure 2d; see SI, Section S5e, for details). This Hf ion surface coverage is clearly less than the maximum possible surface hydroxyl density and fully compatible with a ligand exchange reaction mechanism, in which the proton of the surface hydroxyl is transferred to a DMA^–^ ligand to form DMA and the Hf complex residue binds to the oxygen site.

Exposure to water in the second ALD half-cycle leads to significant quenching of the APXPS signals related to DMA^–^ and MMI. From the O 1s spectra, we find evidence for surface hydroxyls after the reaction with water, and therefore, it seems likely that the DMA^–^ is removed from the surface in a ligand exchange reaction. Such a reaction pathway should not be available for MMI; instead, a hydrolysis reaction seems conceivable^58^ but needs to be investigated further, e.g., by DFT. A hydrolysis reaction of an MMI ligand would liberate a Hf site available for hydroxylation. Depending on the local structure of the Hf site, a hydroxylation reaction could, indeed, be feasible,^59^ and the HfO_2_ surface prepared in the first ALD cycle could be fully hydroxylated. Given the availability of surface hydroxyls, the second, and further, ALD cycles are then expected to follow a ligand exchange reaction mechanism.

## Conclusions

In conclusion, we report an *operando* APXPS experiment, in which we have monitored the first cycle of HfO_2_ ALD on SiO_2_ from TDMAHf and water in real time and at realistic pressure and temperature conditions, i.e., at a precursor pressure typical for ALD growth in a dedicated reactor and a substrate temperature of 280 °C. The measurements allow us to follow the evolution of all relevant core levels and the surface chemical species with a time resolution of 13 s, a value that so far is unprecedented in ALD APXPS experiments. From their spectral fingerprints, we identify DMA^–^ and MMI surface chemical species; the absence of DMA on the surface is ascribed to the relatively high processing temperature above the desorption temperature of DMA. In the water half-cycle, the Hf ions react immediately to HfO_2_, but a significant amount of carbon and nitrogen impurities remains in the film.

The excellent time resolution gives us the possibility to elucidate the order of appearance of the DMA^–^ and MMI surface chemical species. MMI is only formed when a sufficient DMA^–^ coverage is reached on the SiO_2_ surface. We take this as a strong indication for a bimolecular rather than unimolecular character of the decomposition reaction that leads to the formation of MMI. The DFT calculations confirm the dimer formation and that a bimolecular reaction mechanism, based on the insertion of the β-hydride into the bond between one of the Hf ions of the dimer and the N atom of one of the ligands to form DMA, is significantly more favorable than a unimolecular reaction mechanism.

The very initial reaction of TDMAHf on SiO_2_ is, however, based on a ligand exchange mechanism. This reaction cannot take place on the nonhydroxylated stoichiometric parts of the SiO_2_ surface but requires the presence of surface hydroxyls. We postulate that hydroxyls adsorb at defects sites of the SiO_2_ surface and show that the appearance of the O 1s APXP spectrum agrees with the presence of a sufficient number of surface hydroxyls for the ligand exchange reaction to take place.

The subsequent water ALD half-cycle leads to the preparation of a hydroxylated surface. This suggests that further ALD cycles also will proceed according to the ligand exchange reaction scheme. Nonetheless, the present study makes it clear that alternative reaction pathways exist and that alkylamido metal complex ALD also can be carried out on hydroxyl-free surfaces, likely in stark contrast to other ALD metal precursors that would not react with such surfaces.

Finally, our findings also illustrate the potential of APXPS to contribute to a much deepened understanding of the reaction mechanisms in ALD. Such an improved understanding could contribute very significantly to improved ALD processes and processing parameters and thus to an improved quality of thin films grown by ALD. While the experiments presented here were carried out using exposure of SiO_2_ to the precursors that were extended in comparison to typical ALD pulse durations, recent development of the time-resolved capabilities of the APXPS methodology for periodic processes such as ALD, at retained or even improved signal-to-noise ratios,^60,61^ suggests that in the future, it will become possible to perform ALD APXPS experiments under truly realistic ALD reactor conditions, in terms of the pressure, temperature, and pulse duration. In the future, it may even become possible for ALD experiments to combine the recording of simultaneous, time-resolved APXPS and X-ray scattering data,^62^ thus allowing us to obtain complementary chemical and structural information.

## Supporting Information Available

(S1) Gas delivery system, (S2) comparison of pressure conditions in the present study and standard ALD experiments, (S3) calculations methods, (S4) data treatment and curve fitting, (S5) interpretation of the N 1s:Hf 4f intensity ratio and determination of the surface coverage, (S6) interpretation of the C 1s:N 1s intensity ratio, (S7) DFT results and decomposition of TDMAHf, and (S8) kinetic modeling (PDF)
