# Supplementary material for: Bimolecular Reaction Mechanism in the Amido Complex-Based Atomic Layer Deposition of HfO2
Source: Chem Mater. 2023 Jan 3;35(2):529–38. doi: 10.1021/acs.chemmater.2c02947 (PMC9879184; doi:10.1021/acs.chemmater.2c02947)
Supplement: Supplementary file 1 — cm2c02947_si_001.pdf [file cm2c02947_si_001.pdf]

Bimolecular reaction mechanism in the amido complex-based atomic layer deposition of HfO_2

Giulio D'Acunto,[a,†] Roman Tsyshevsky,[b,†] Payam Shayesteh,[a,†] Jean-Jacques Gallet,[c,d] Fabrice Bournel,[c,d] François Rochet,[c] Indiana Pinsard,[a] Rainer Timm,[a] Ashley R. Head,[e] Maija Kuklja,[b] Joachim Schnadt[a,f,]*

[a] Lund University, Department of Physics, Division of Synchrotron Radiation Research, and NanoLund, Box 118, 221 00 Lund, Sweden

[b] University of Maryland, Department of Materials Science and Engineering, College Park, Maryland 20742, United States of America

[c] Sorbonne Université, CNRS Laboratoire de chimie physique-Matière et rayonnement, 4 place Jussieu, 75005 Paris, France

[d] Synchrotron SOLEIL, L'Orme des Merisiers, Saint-Aubin, BP 48, 91192 Gif-sur-Yvette Cedex, France

[e] Brookhaven National Laboratory, Center for Functional Nanomaterials, P.O. Box 5000, Upton, New York 11973-5000, United States of America

[f] Lund University, MAX IV Laboratory, Box 118, 221 00 Lund, Sweden

[*] E-mail: joachim.schnadt@sljus.lu.se

[†] These authors have contributed equally to the manuscript.

Supporting Information

S1. Gas delivery system

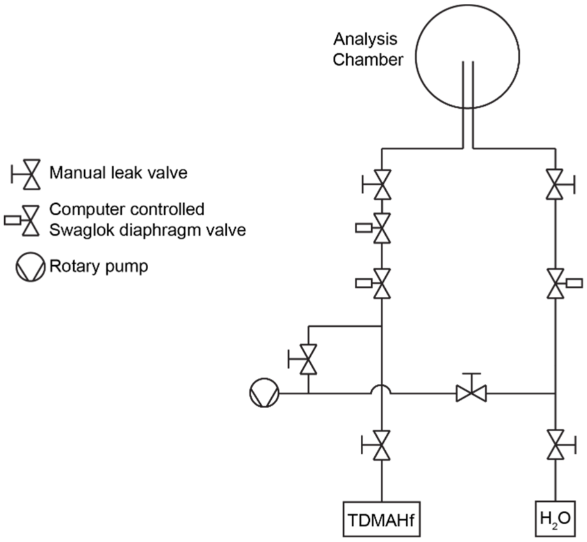

Figure S1. Sketch of the ALD dosing system. The distance between the two inlets and the sample was around 3cm. The entire system was heated to 60 °C. During the deposition of the first precursor, the gas line of the second one was pumped constantly, and vice versa.

S2. Comparison of pressure conditions in the present study and standard ALD experiments

It is of relevance to compare the pressure conditions of the APXPS ALD experiment to those in standard ALD experiments in ALD reactors. In these latter experiments the pressure during the ALD pulse is not well known. For HfO_2 ALD from TDMAHf a few studies, however, state the metal precursor pressure during the pulse;[S1-S8] the pressures are found to vary between 1×10^{-3} and 2.7 mbar. Other studies[S9-S23] state the TDMAHf temperature, which allows one to estimate the TDMAHf vapor pressure using the parameters of Hausmann *et al.*[S9] and the Clausius-Clapeyron relation. The thus estimated vapor pressure with values in between 1×10^{-3} and 1.3 mbar is an upper limit for the working pressure. The bulk of work on HfO_2 ALD from TDMAHf seems have to be carried out at metal precursor peak pressure of around 0.6 mbar, i.e. at a pressure that is around 30 times higher than the pressure that we used in our experiment.

S3. Calculation methods

All molecular calculations were performed with DFT[S24] using the hybrid M06-2X[S25] functional as implemented in the Gaussian 09 code.[S26] A double-ζ 6-31G(d,p) basis set was used for the H, C, and N atoms. The LanL2DZ basis set,[S27-S29] including the Los Alamos effective core potential, was used for the Ti and Hf atoms.

Solid-state periodic calculations were performed by employing DFT with the optPBE-vdW[S30-S34] functional, which includes corrections for van der Waals interactions, as implemented in the VASP code.[S35-S37] The projector-augmented wave (PAW) method[S38] was used. Solid-state calculations were carried out on the oxidized Si(111)-(7×7) and a SiO_2(001) surface (Figure S2).

In calculations of a Si bulk crystal (Figure S2a), the convergence criterion for the total energy was set to 10^{-5} eV, and the maximum force acting on each atom in the periodic supercell was set not to exceed 0.02 eV/Å. A 12×12×12 Monkhorst−Pack *k*-point mesh with a kinetic energy cut-off of 520 eV was used. The optimized lattice constants of the cubic unit cell were $a = b = c = 5.47$ Å, which agree with the experimentally determined ones ($a = b = c = 5.43$ Å)[S39] to within 1%. The Si(111)-(7×7) surface, which is the most well-understood silicon surface,[S40] was simulated by a periodic (2×2) supercell containing 168 atoms and with the lattice parameters $a = b = 15.48$ Å and $c = 38.17$ Å (Figure S2b). The supercell contained four adatoms and four rest atoms. An oxide layer was introduced on the Si (111)-(7×7) surface introduced by adding four oxygen atoms to each Si adatoms (Figure S2c) as described in Ref. S41. A vacuum layer of 20 Å placed on top of the Si(111) surface served to minimize the interactions between the supercells in the *z*-direction and to avoid any significant overlap between electron density of periodically translated cells. Surface supercell calculations were performed at the Γ point only, with the convergence criteria for electronic and ionic steps set to 10^{-5} eV and 0.03 Å/eV, respectively.

In calculations of a stoichiometric SiO_2 crystal (Figure S2d), the convergence criterion for the total energy was set to 10^{-5} eV, and the maximum force acting on each atom in the periodic supercell was set not to exceed 0.02 eV/Å. The 10×10×10 Monkhorst−Pack *k*-point mesh with a kinetic energy cut-off of 520 eV was used. The optimized lattice constants of the hexagonal unit cell were $a = b = 4.93$ Å, $c = 5.45$ Å, agreeing with the experimentally determined ones[S42] to within 1%. In the SiO_2 surface calculations (Figure S2e), the model surface slab was cut out of the bulk SiO_2 structure to form the (001) surface, with the supercell lattice vectors of a =19.71 Å, b =17.07 Å and c = 37.63 Å. A vacuum layer of 20 Å placed on top of the SiO_2(001) surface served to minimize the interactions between the supercells in the *z*-direction and to avoid any significant overlap between electron density of periodically translated cells. The structure of the reconstructed SiO_2(001) surface was adopted from Ref. S43. Surface supercell calculations were performed at the Γ point only, with the convergence criteria for electronic and ionic steps set to 10^{-5} eV and 0.03 Å/eV, respectively.

Minimum energy paths in the VASP periodic calculations were obtained with the nudged elastic band method.[S44] Atomic positions of the intermediate images were relaxed using the conjugate gradient and quasi-Newtonian methods within a force tolerance of 0.05 eV/Å.

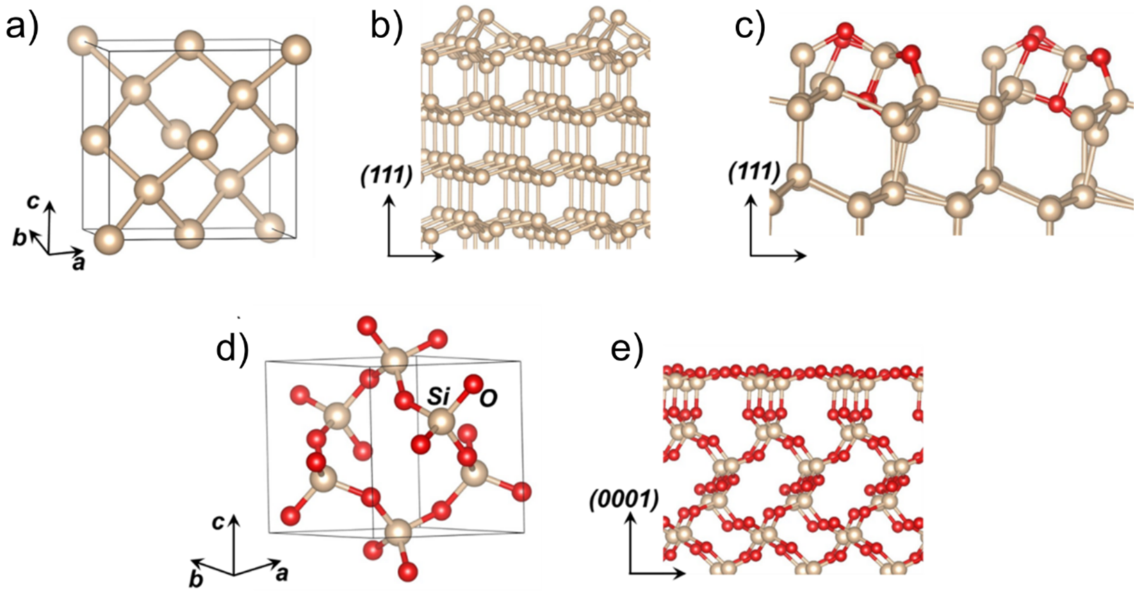

Figure S2. Structure of a) bulk Si crystal, b) Si (111)-(7×7) surface, and c) Si (111)-(7×7) surface with oxide layer, (d) bulk SiO_2 crystal, (e) SiO_2(001) surface

S4. Data treatment and curve fitting

The experimental data were analyzed using the Igor Pro software by Wavemetrics Inc. A constant background was subtracted from all APXP spectra recorded in snapshot mode. Least-square curve fitting was carried out using Gaussian profiles using a global fit procedure, i.e. all the spectra displayed in a single image plot [e.g. in Fig. 1(b)] were fitted simultaneously. Components assigned to the same type of atomic species were forced to have the same binding energy and the same Gaussian width. These two parameters were, however, allowed to vary globally. The components' amplitudes were completely free parameters, with one exception: since MMI contains both a methyl group (with a C 1s spectral signature that was assumed to have the same binding energy as that of the methyl groups of DMA^-) and a methylene group, the methyl and methylene intensities related to MMI were forced to have the same intensity. The N 1s and Hf 4f gas phase spectra of TDMAHf in Fig. S3 were curve-fitted with Voigt profiles after removal of a Shirley background.

S5. Interpretation of the N 1s:Hf 4f intensity ratio and determination of the surface coverage

Fig. 2(a) displays the N 1s:Hf 4f intensity ratio during the first ALD half-cycle and, derived from this ratio, the number of ligands retained in the surface-adsorbed –Hf(DMA^-)$_x$ complexes, Fig. 2(c) the surface coverage with –Hf(DMA^-)$_x$ surface complexes and Fig. 2(d) the corresponding surface density. The present section details how these curves were derived from the experimental data. It is divided into four parts: Section S5a is concerned with the composition of the TDMAHf vapor, which contained both intact and dissociated TDMAHf molecules and section S5b with the interpretation of the N 1s:Hf 4f intensity ratio of the gas phase signal. The results of these two sections are then used to interpret the N 1s:Hf 4f intensity ratio of the surface-adsorbed complexes in section S5c. The section makes also use of the results of section S5d, in which we describe how we treated the attenuation of the photoelectrons in the photoemitting compounds. Finally, in section S5e it is described how we estimated the density of surface hydroxyls on the SiO_2 surface.

S5a. TDMAHf vapor composition

The TDMAHf vapor composition is estimated from the gas phase N 1s and Hf 4f core-level spectra, obtained in 0.03 mbar TDMAHf. The data are shown in Figure S3. Clearly, the N 1s line is composed of two components, centered at 403.0 and 404.8 eV.[S45] The dominant one, with an intensity I_{DMA^-}, is associated with Hf-bonded DMA^-, while the high-binding energy shoulder with an intensity I_{DMA} is related to free DMA. The DMA component accounts for 10 % of the total N 1s intensity. It is noted that the observed N 1s and Hf 4f binding energies are several eV larger than the corresponding core levels of the surface-adsorbed core levels (cf. Fig. 1). The difference is due to the much improved core-hole screening in the final state of photoemission provided by the solid Si substrate.

The free DMA necessarily stems from dissociation of TDMAHf complexes. We consider it most likely that DMA is formed in hydrolysis reactions of gas-phase TDMAHf complexes with residual water vapor, with hydroxy-tris(dimethylamido) hafnium ($Hf(N(CH_3)_2)_3OH$) as the second reaction product. Our assumption is based on the exothermicity and low activation energy of the vapor hydrolysis of amido Hf complexes, which is easily overcome at room temperature.[S46] Furthermore, a sufficient background pressure of residual water is present in essentially any vacuum system operated in the 10^{-2} mbar (or higher) region. It should be noted that the DMA cannot result from dissociation of TDMAHf adsorbed on the pipe and chamber walls since its desorption temperature is significantly higher than room temperature.[S47]

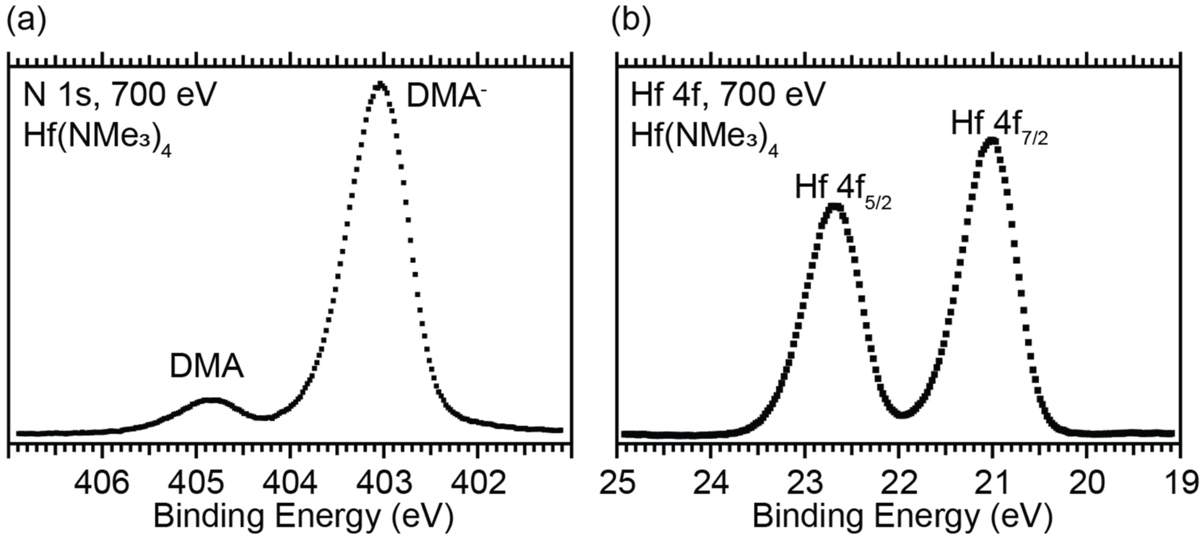

Figure S3. Gas phase (a) N 1s and (b) Hf 4f XP spectra measured at a pressure of 0.03 mbar at 700 eV photon energy.

The measured ambient pressure photoemission intensities of a particular core-level line can generally be expressed as $I = n \cdot N \cdot I_0 \cdot C \cdot \sigma \cdot T \cdot C_g$. Here, n is the numbers of the photoemitting compounds in the volume probed by the x-ray beam, or, alternatively, the compound's fraction of the total number of molecules. N is the number of photoemitting atoms (of a particular element and thus core level) in the compound. I_0 is the energy-resolved and photoionization cross section-

corrected photocurrent for the particular core level into the solid angle of the electron energy analyzer; I_0 is described by the squared transition matrix element in the dipole approximation,[S48] C is a factor that describes the attenuation of the photoelectrons in the photoemitting compounds. An estimation of the relevant attenuation factors is provided in section S5d. σ is the subshell photoionization cross section, T the kinetic energy-dependent analyzer transmission and C_g the kinetic energy-dependent attenuation in the gas phase. σ, T and C_g are taken to be the same for all photoemission events of a particular core level. This is justified by the use of the same photon energy (and hence the same kinetic energy for a particular core level) in all measurements.

We expect the XPS signals of the DMA$^-$ ligands of $Hf(N(CH_3)_2)_3OH$ and $Hf(N(CH_3)_2)_4$ to be indistinguishable from each other, and therefore $I_{N1s}^{DMA^-} = I_{N1s}^{3\,DMA^-} + I_{N1s}^{4\,DMA^-}$, where $I_{N1s}^{4\,DMA^-}$ and $I_{N1s}^{3\,DMA^-}$ denote the N 1s photoemission intensities related to $Hf(N(CH_3)_2)_4$ (with four DMA$^-$ ligands) and $Hf(N(CH_3)_2)_3OH$ (with three DMA$^-$ ligands), respectively. Using all of the above, the total N 1s photoemission intensity is written as

$$I_{N1s} = I_{N1s}^{DMA} + I_{N1s}^{DMA^-} = I_{N1s}^{DMA} + I_{N1s}^{3\,DMA^-} + I_{N1s}^{4\,DMA^-} = \\ (n_{DMA} \cdot N_{DMA} \cdot I_{N1s;0}^{DMA} \cdot C_{DMA} + n_{3\,DMA^-} \cdot N_{3\,DMA^-} \cdot I_{N1s;0}^{3\,DMA^-} \cdot C_{3\,DMA^-} \\ + n_{4\,DMA^-} \cdot N_{4\,DMA^-} \cdot I_{N1s;0}^{4\,DMA^-} \cdot C_{4\,DMA^-}) \cdot \sigma_{N1s} \cdot T_{N1s} \cdot C_{g;N1s} \quad (1)$$

Here, we assume in first approximation that these matrix elements are the same irrespective of the photoemitting atoms' surroundings, i.e. $I_{N1s;0} \equiv I_{N1s;0}^{DMA} = I_{N1s;0}^{3\,DMA^-} = I_{N1s;0}^{4\,DMA^-}$. $N_{DMA} = 1$ ($N_{3\,DMA^-} = 3$; $N_{4\,DMA^-} = 4$) is the number of nitrogen atoms in DMA (in the $Hf(N(CH_3)_2)_3OH$ complex; in the $Hf(N(CH_3)_2)_4$ complex).

As already stated, the DMA intensity accounts for 10 % of the total N 1s intensity. Since $Hf(N(CH_3)_2)_3OH$ and DMA result from the same reaction in equal quantities, $n_{3\,DMA^-} = n_{DMA}$, and hence:

$$\frac{I_{DMA}}{I_{DMA} + I_{3\,DMA^-} + I_{4\,DMA^-}} = 0.1 =$$

$$= \frac{n_{3\,DMA^-} \cdot N_{DMA} \cdot C_{DMA}}{(n_{3\,DMA^-} \cdot N_{DMA} \cdot C_{DMA} + n_{3\,DMA^-} \cdot N_{3\,DMA^-} \cdot C_{3\,DMA^-} + n_{4\,DMA^-} \cdot N_{4\,DMA^-} \cdot C_{4\,DMA^-})}. \quad (2)$$

In order to estimate the average number of ligands in the vapor hafnium complexes that interact with the SiO_2 surface, we take the ns to be fractions and normalize to the number of Hf complexes: $n_{3\,DMA^-} + n_{4\,DMA^-} = 1$. From the preceding equation, with the estimates for the C factors form section S5d, we then find $n_{3\,DMA^-} = 35\,\%$ and $n_{4\,DMA^-} = 65\,\%$. The average number of ligands in the vapor hafnium complexes is then found to be

$$\bar{N}_{lig} = N_{3\,DMA^-} \cdot n_{3\,DMA^-} + N_{4\,DMA^-} \cdot n_{4\,DMA^-} = 3.65. \quad (3)$$

S5b. Gas phase N 1s:Hf 4f intensity ratio

Using the same nomenclature as in section S5a, the gas phase intensity ratio of the DMA^- N 1s component to the Hf 4f line can be written as

$$\frac{I_{N1s,g}^{DMA^-}}{I_{Hf4f,g}} =$$

$$= \frac{(n_{3\,DMA^-} \cdot N_{N.3\,DMA^-} \cdot C_{N,3\,DMA^-} + n_{4\,DMA^-} \cdot N_{N,4\,DMA^-} \cdot C_{N,4\,DMA^-})}{(n_{3\,DMA^-} \cdot N_{Hf,3\,DMA^-} \cdot C_{Hf,3\,DMA^-} + n_{4\,DMA^-} \cdot N_{Hf,4\,DMA^-} \cdot C_{Hf,4\,DMA^-})} \cdot$$

$$\cdot \frac{I_{N1s;0} \cdot \sigma_{N1s} \cdot T_{N1s} \cdot C_{N1s,g}}{I_{Hf4f;0} \cdot \sigma_{Hf4f} \cdot T_{Hf4f} \cdot C_{Hf4f,g}}. \quad (4)$$

The T and C_g factors are generally unknown, but characteristic for the chosen photon energy and, hence, kinetic energy. Therefore, we introduce two new parameters, characteristic for each core level:

$$a_{N1s} = I_{N1s;0} \cdot \sigma_{N1s} \cdot T_{N1s} \cdot C_{N1s,g}, \quad (5)$$

$$a_{Hf4f} = I_{Hf4f;0} \cdot \sigma_{Hf4f} \cdot T_{Hf4f} \cdot C_{Hf4f}. \quad (6)$$

With the other parameters as in section S5a and S5d, we find

$$\frac{I_{N1s,g}^{DMA^-}}{I_{Hf4f,g}} = \frac{2.505 \cdot a_{N1s}}{0.798 \cdot a_{Hf4f}} = 3.140\ \frac{a_{N1s}}{a_{Hf4f}}. \quad (7)$$

We can now equate eq. (7) with the experimental value for the DMA^- N 1s:Hf 4f intensity ratio in the core level spectra shown in Fig. S3. We obtain:

$$\frac{a_{N1s}}{a_{Hf4f}} = \frac{0.58}{3.140} = 0.184. \quad (8)$$

S5c. Surface N 1s:Hf 4f intensity ratio, number of ligands per surface-adsorbed Hf complex and molecular coverage

In this section we consider the surface N 1s:Hf 4f intensity ratio I_{N1s}/I_{Hf4f} (Fig. 2a, blue line) during the initial exposure of the oxidized Si(111) surface to TDMAHf, until appearance of the MMI signal. The intensity ratio provides information on the average number of DMA^- ligands retained per surface-adsorbed Hf complex (Fig. 2a, red dots), and derive the Hf complex surface coverage (Fig. 2c). In our treatment, we assume that gas phase DMA does not adsorb on the surface and that it therefore does not affect the intensity of the surface photoemission lines. The assumption is justified by the substrate temperature of 280 °C, well above the DMA desorption temperature.[S47] We also assume that further hydrolysis, beyond that discussed in section S5a, does not occur and that the hydroxylated gas phase complexes react with the SiO_2 surface alongside the fully intact TDMAHf complexes.

The gas phase complexes TDMAHf, with four DMA^- ligands, and $Hf(N(CH_3)_2)_3OH$, with three DMA^- ligands, can both adsorb on the SiO_2 surface. As the DFT calculations show, TDMAHf physisorbs on the stoichiometric surface in intact form, i.e. with four DMA^- ligands retained. We assume that ligand exchange reactions are possible at defect sites with adsorbed surface hydroxyls. It seems sterically unlikely that a favorable adsorption geometry with three retained DMA^- ligands can be found, and therefore we assume that the reaction is always towards a surface complex with

two DMA$^-$ ligands. In such a reaction, two surface hydroxyls are consumed for adsorption of TDMAHf. With respect to $Hf(N(CH_3)_2)_3OH$, a direct reaction between the surface and the hydroxyl group of the complex seems feasible. In such a reaction the hydroxyl's proton is assumed to form DMA together with another of the DMA$^-$ ligands, yielding the same surface Hf complex with two retained DMA$^-$ ligands as in the ligand-exchange reaction of TDMAHf with hydroxyls at defect sites. Hence, we assume that two types of surface Hf complexes exist in the early stages of TDMAHf exposure: physisorbed TDMAHf with four DMA$^-$ ligands and surface-O_2-Hf-$(N(CH_3)_2)_2$ complexes.

S5c.1 Surface N 1s:Hf 4f intensity ratio, number of ligands per surface-adsorbed Hf complex

The N 1s to Hf 4f intensity ratio I_{N1s}/I_{Hf4f} can the be written as follows:

$$\frac{I_{N1s}^{DMA^-}}{I_{Hf4f}} =$$

$$= \frac{\left(n_{2\,DMA^-} \cdot N_{N.2\,DMA^-} \cdot C_{N,2\,DMA^-} + n_{4\,DMA^-} \cdot N_{N,4\,DMA^-} \cdot C_{N,4\,DMA^-}\right) \cdot \alpha_{N1s}}{\left(n_{2\,DMA^-} \cdot N_{Hf,2\,DMA^-} \cdot C_{Hf,2\,DMA^-} + n_{4\,DMA^-} \cdot N_{Hf,4\,DMA^-} \cdot C_{Hf,4\,DMA^-}\right) \cdot \alpha_{Hf4f}}. \quad (9)$$

The α factors are the same as for the gas phase measurements, which is justified by the use of the same photon energy (and hence kinetic energies) in the gas phase and surface measurements. $n_{2\,DMA^-}$ is the number of surface-O_2-Hf-$(N(CH_3)_2)_2$ complexes in the volume probed by the x-rays and $N_{N.2\,DMA^-}$=2. With $\frac{a_{N1s}}{a_{Hf4f}}$ as in eq. (8) and the *N* and *C* parameters as in sections S5a and S5d, eq. (9) becomes

$$\frac{I_{N1s}^{DMA^-}}{I_{Hf4f}} = 0.184\frac{1.490\,n_{2\,DMA^-} + 2.712\,n_{4\,DMA^-}}{0.793\,n_{2\,DMA^-} + 0.793\,n_{4\,DMA^-}}. \quad (10)$$

If we again impose a normalization condition

$$n_{2\,DMA^-} + n_{4\,DMA^-} = 1, \quad (11)$$

we can solve eq. (10) using the experimental N 1s to Hf 4f intensity ratios derived from the spectra in Fig. 1 to give the fractions $n_{2\,DMA^-}$ and $n_{4\,DMA^-}$ of surface-adsorbed complexes with two and four retained ligands, respectively. The average number of ligands per surface-adsorbed complex is then

$$\bar{N}_{lig}(t) = n_{2\,DMA^-}(t) \cdot N_{2\,DMA^-} + n_{4\,DMA^-}(t) \cdot N_{4\,DMA^-}. \tag{12}$$

For example, at $t^* = 0$, which we define as the time of the first occurrence of the Hf 4f and N 1s intensities related to TDMAHf and $Hf(N(CH_3)_2)_3OH$ adsorption, we find $\left. I_{N1s}^{DMA^-} \middle/ I_{Hf4f} \right|_{t^*} = 0.42$, which gives $n_{2\,DMA^-}$=74 % and $n_{4\,DMA^-}$= 26 %, and $\left.\bar{N}_{lig}\right|_{t^*} = 2.51$. We estimate the uncertainty of $\bar{N}_{lig}$ to be around 0.35.

S5c.2 Molecular coverage

The molecular coverage $\vartheta(t)$, specified in terms of fraction of a single fully saturated molecular layer adsorbed on the oxidized Si(111) surface, can be estimated from the intensity evolution of the Si 2p core level as a function of time as follows:

$$\frac{I_{Si2p}(t)}{I_{0;Si2p}(t=0)} = \vartheta(t)e^{-\frac{d(t)}{\lambda}} + \left(1 - \vartheta(t)\right). \tag{13}$$

$I_{Si2p}(t)$ is the intensity of the Si 2p core level during the exposure, $I_{0;Si2p}$the intensity of the Si 2p line before the exposure, λ=20 Å the attenuation length, $d(t)$ the thickness of the overlayer and $\vartheta(t)$ the coverage as a function of exposure time.

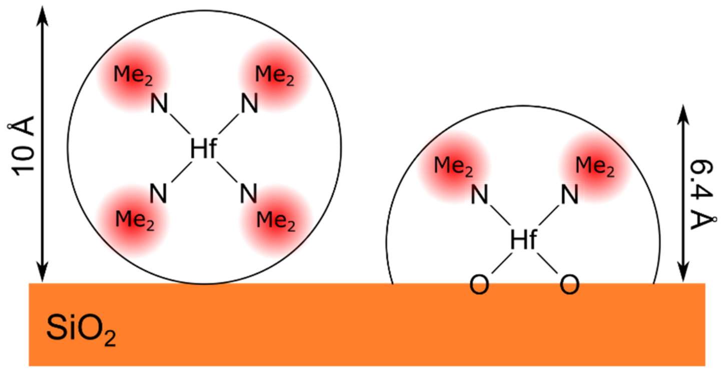

Figure S4. Illustration of the model used in the estimation of the thickness of the molecular overlayer.

In eq. (13), most of the parameters are known, except for *d(t)* and $\vartheta(t)$. *d(t)* is determined by the height of the surface-adsorbed TMDAHf and surface-O_2-Hf-($N(CH_3)_2)_2$ complexes (Fig. S4). For TDMAHf this height is, in accordance with section S5d.1, 10 Å, i.e. twice the approximate radius of the TDMAHf complex For the surface-O_2-Hf-($N(CH_3)_2)_2$ complexes we estimate the height to be 6.4 Å. This is done using the geometry described in section S5d.3, under the assumption that the Hf-O bond length is the same as the Hf-N in TDAMHf and that the Hf-N bond lengths are unchanged in comparison to TDMAHf. From eq. (10) we estimate the fraction of surface complexes with two or four retained ligands, respectively, and can then derive the average thickness as a function of time $d(t)$ according to

$$d(t) = n_{2\,DMA^-}(t) \cdot 6.4\,\text{Å} + n_{4\,DMA^-}(t) \cdot 10\,\text{Å}. \tag{14}$$

Knowledge of $d(t)$ allows solving eq. (13) to derive $\vartheta(t)$.

The molecular coverage can also be expressed in terms of the adsorbate density. We assume a hexagonal close-packed arrangement of the spheres representing the TDMAHf and surface-O_2-Hf-($N(CH_3)_2)_2$ surface complexes, both with a diameter parallel to the surface of $D = 10$ Å. For a hexagonal close-packed layer the density of a full monolayer can be calculated from

$$\rho_{complexes} = \frac{3}{3 \cdot \frac{\sqrt{3}}{2} \cdot D^2}. \tag{15}$$

A full monolayer corresponds to 0.0115 complexes/Å^2. The density of the complexes with only two retained DMA$^-$ ligands can then be estimated from $n_{2\,DMA^-}(t)$. This surface density of surface-adsorbed complexes with two retained DMA$^-$ ligands, formed in ligand-exchange reactions, is shown in Figure 2d.

S5d. Attenuation of the photoelectrons in the photoemitting compounds

The photoemission signal is affected by attenuation in the photoemitting compounds, irrespective of whether the photoemission event takes place in the vapor phase or in surface-adsorbed compounds. In this section, we estimate the corresponding attenuation factors, using characteristic attenuation lengths λ in organic materials[S49] of 12.0 Å for N 1s photoemission ($h\nu$=700 eV, i.e. the kinetic energy is approximately 300 eV) and 21.5 Å for Hf 4f photoemission (same photon energy and therefore a kinetic energy of approximately 680 eV).

S5d.1 Internal attenuation of the N 1s signal in TDMAHf

To estimate the internal attenuation of the N 1s photoelectrons in a TDMAHf molecule, we approximate the TDMAHf complex by a sphere, with a radius defined by the distance between the Hf ion and the outward-pointing H atoms plus the H van der Waals radius (1.2 Å). The coordinates of the atoms in the TDMAHf complex stem from a DFT calculation published previously.[S45] We find a radius of the sphere of 5 Å. The distance that the N 1s photoelectrons travel inside the sphere can then be calculated from the coordinates $x_{N_i}, y_{N_i}, z_{N_i}$ $(i = 1 \ldots 4)$ of the N atoms in the sphere. Assuming that the analyzer axis is oriented along the z axis, we can we calculate the photoelectron travel distance for each N atom as $d_i = |z - z_{N_i}| = \sqrt{r^2 - y_{N_i}^2 - x_{N_i}^2} - z_{N_i}$. After averaging over all possible orientations of the TDMAHf molecule with respect to the analyzer we find an attenuation factor

$$C_{N,4\,DMA^-} = \frac{1}{N^3} \sum_{\alpha,\beta,\gamma=0}^{360} \left(\frac{1}{4} \cdot \sum_{i=1}^{4} e^{-\frac{d_i(\alpha,\beta,\gamma)}{\lambda}} \right) = 0.678, \tag{16}$$

where α, β and γ indicate the angles of rotation around the *x*, *y* and *z* axes, respectively, and *N* the number of summands. $d_i(\alpha, \beta, \gamma)$ was calculated at steps 10° and, hence $N = 36$.

The same averaging over all the possible orientation is used in the calculations in S5d.2.

S5d.2 Internal attenuation of the N 1s signal in $Hf(N(CH_3)_2)_3OH$

To find an estimate for the attenuation of the photoelectrons in $Hf(N(CH_3)_2)_3OH$ we assume that the volume from where the photoelectrons escape is composed two half spheres, one containing the three methyl groups and the other the hydroxyl group. Further, we assume that the radius of the volume containing the methyl groups is the same of that used in the estimation of the attenuation in TDMAHf mol, i.e. the radius is 5 Å. The radius of the hydroxyl-containing half-sphere is estimated from the Hf-O and O-H distances together with the hydrogen van der Waals radius (1.20 Å). Here, the Hf-O distance is taken to be equal to the Hf-N distance in TDMAHf (2.04 Å), and the O-H distance is 0.97 Å.[S45] Hence, we find a radius for the smaller half-sphere of 4.2 Å. The average radius of the two half-sphere is 4.6 Å, and, given that the photoemitting $Hf(N(CH_3)_2)_3OH$ can have any orientation towards the analyzer, we use a sphere with this average radius to estimate the photoelectron attenuation in the hydroxyl complex:

$$C_{N,3\,DMA^-} = \frac{1}{N^3} \sum_{\alpha,\beta,\gamma=0}^{360} \left(\frac{1}{3} \cdot \sum_{i=1}^{3} e^{-\frac{d_i(\alpha,\beta,\gamma)}{\lambda}} \right) = 0.703. \tag{17}$$

S5d.3 Internal attenuation of the N 1s signal in surface-bonded $Hf(N(CH_3)_2)_2$ complexes

For the configuration of the surface-adsorbed complexes with two retained ligands, we assume that the ligands always are oriented away from the surface in a symmetric fashion and that the volume from where the N photoelectron escape as a half sphere oriented towards the analyzer. Since the angle between the two nitrogen-hafnium bond is θ =109.5° and the bond length 2.04 Å, we find the coordinates of the two nitrogen atoms to be $\left(x_{N_i}, y_{N_i}, z_{N_i}\right) = 2.04 \cdot (\sin(\theta), 0, \cos(\theta))$. As before $d_i = \left|z - z_{N_i}\right| = \sqrt{r^2 - y_{N_i}^2 - x_{N_i}^2} - z_{N_i}$ and, therefore,

$$C_{N,2\,DMA^-} = \frac{1}{2} \cdot \sum_{i=1}^{2} e^{-\frac{d_i}{\lambda}} = e^{-\frac{d}{\lambda}} = 0.745, \tag{18}$$

since the two distances d_i are the same ($d = 3.438$ Å).

S5d.4 Internal attenuation of the N 1s signal in DMA

We treat the attenuation in DMA in a similar way as that in $Hf(N(CH_3)_2)_3OH$. We assume that the distribution of the methyl groups is uniform and that they are contained in a half-sphere. Another half-sphere contains the C-bonded hydrogen atom. With a N-C bond length of 1.46 Å, a C-H bond length of 1.09 Å, an NCH angle of 109° and a van der Waals radius of H of 1.20 Å, the methyl-containing half-sphere has a radius of 3.29 Å. The hydrogen atom-containing half sphere has a radius of 2.22 Å, derived from the N-H distance of 1.02 Å and the H van der Waals radius. As for $Hf(N(CH_3)_2)_3OH$ the photoemitting DMA can have any orientation towards the analyzer and we therefore assume that the photoelectrons on average travel through a sphere with radius that is the average of the two radii of the half-sphere (d=2.76 Å). We then find for the internal photoelectron attenuation in DMA:

$$C_{N,DMA} = e^{-\frac{d}{\lambda}} = 0.795. \tag{19}$$

S5d.5 Internal attenuation of the Hf 4f signal in TDMAHf

The estimate is derived in the same was as for the N 1s signal in TDMAHf in section S5d.1, but with the Hf ion residing in the center of the molecule, i.e. the photoelectron travel distance is 5 Å. Therefore

$$C_{Hf,4\,DMA^-} = e^{-\frac{d}{\lambda}} = 0.792. \tag{20}$$

S5d.6 Internal attenuation of the Hf 4f signal in $Hf(N(CH_3)_2)_3OH$

Using the same geometry as in section S5d.2 and having the Hf ion in the center of the sphere (average radius d=4.6 Å), we find

$$C_{Hf,3L} = e^{-\frac{d}{\lambda}} = 0.807. \tag{21}$$

S5d.7 Internal attenuation of the Hf 4f signal in surface-bonded $Hf(N(CH_3)_2)_2$ complexes

Finally, in the surface-adsorbed $Hf(N(CH_3)_2)_2$ complexes the Hf ion is bonded to the surface and the two remaining DMA^- ligands point outwards the surface. The attenuation of the Hf 4f photoelectron is the same as for TDMAHf (section S5d.5) and

$$C_{Hf,2\,DMA^-} = C_{Hf,4\,DMA^-} = e^{-\frac{d}{\lambda}} = 0.792. \tag{22}$$

S5d.8 Summary of the factors describing the internal attenuation of the photoelectrons in the photoemitting compounds

The attenuation factors derived in sections S5d.1 to S5d.7 and S6c are summarized in Table S1.

	TDMAHf	$Hf(N(CH_3)_2)_3OH$	Surface-bonded *$Hf(N(CH_3)_2)_2$*	DMA
C_{Hf}	0.792	0.807	0.792	//
C_N	0.678	0.703	0.745	0.795
C_C	0.744	0.770	0.909	0.842
C_N/C_{Hf}	0.856	0.871	0.94	//

Table S1. Summary of the factors that describe the internal attenuation of the photoelectrons in the different photoemitting compounds.

S5e. Interpretation of the O 1s line and hydroxyl coverage

S5e.1 Interpretation of the O 1s spectrum of the oxidized Si(111)-(7×7) surface

Oxidation of the Si(111)-(7×7) reconstruction, as mirrored by the appearance of the O 1s spectrum of the oxidized surface (Fig. 1), involves primarily the so-called Si adatoms,[S50] but also the Si "restatoms" may be involved at higher oxygen coverage. At room temperature, and given sufficient time for relaxation, the addition of oxygen to the surface leads to the formation of *ins*×*n* and *ins*×*n-ad* structures (*n* O_{ins} oxygen atoms inserted into the bonds between the Si adatom and neighboring Si atoms and, in the case of the *ins*×*n-ad* structure, an additional oxygen adatom on top of the Si adatom).[S41,S51] Upon annealing the *ins*×*n-ad* structure is converted into an *ins*×*n-tri* structure, with the oxygen adatom moved into a subsurface (*tri*) site, where it is bonded to three Si atoms.[S51] For the fully oxidized surface at monolayer oxygen coverage, each Si adatom binds to three O_{ins} atoms, i.e. *n*=3. The Si(111)-(7×7) supercell has a rhombus shape with a side length of 2.688 nm, internal angles of 60 and 120° and thus an area of 625.7 Å^2. Since the supercell contains 12 Si adatoms and thus 36 O_{ins} atoms, it follows that the density of O_{ins} atoms at monolayer oxygen coverage is 0.0575 Å^{-2}. The actual oxygen coverage in our experiment was, however, somewhat higher. We used the same preparation recipe as that used in Ref. S52, known to lead to a coverage of (1.40±0.05)

monolayers. At this somewhat higher coverage also the so-called Si restatoms are oxidized in a manner similar to that of the Si adatoms, i.e. we assume that Si restatom-bonded O_{ins} contribute to the O_{ins} O 1s line. The O_{ins} coverage becomes $1.40 \cdot 0.0575$ Å^{-2} = 0.0805 Å^{-2}.

The appearance of the O 1s spectra in Fig. 1 seemingly agrees with this interpretation: the two O 1s components can be assigned to the *ins* and *tri* oxygen species.[S52] However, the hydroxyl O 1s component on SiO_2 is found at a binding energy that is very close to that of the O_{tri} component, and, hence, the shoulder towards high binding energy could also be associated with surface hydroxyls adsorbed a t surface defects.[S53,S54] Since we do not know to which extent *ins×3-tri* structures have been formed at the surface, we cannot unambiguously determine how much of the high binding-energy peak in the O 1s spectrum is due to O_{tri} species and how much is due to surface hydroxyls.

We can, however, consider the limits of O_{tri} formation: if the surface only contains *ins×3*, but no *ins×3-tri* structures, we expect an O_{ins}:O_{tr} intensity ratio of 3:0. If, on the contrary, only *ins×3-tri*, but no *ins×3* structures are found at the surface, the O_{ins}:O_{tr} intensity ratio would be 3:1. From the first eight spectra in the time-resolved O 1s APXPS data, taken before the first TDMAHf exposure, we find an average O_{ins}:(O_{tr}+OH) intensity ratio of 10:1, in between the two limiting values.

S5e.2 Maximum surface hydroxyl coverage

The experimental O_{ins}:(O_{tr}+OH) intensity ratio in the O 1s line of the clean oxidized surface is 10:1. From the above this implies that the total density of O_{tr} and hydroxyl species is approximately 0.008 Å^{-2}. This is also the maximum possible hydroxyl coverage of the surface, in a situation in which no O_{tr} has been formed.

S5e.3 Comparison of the maximum surface hydroxyl coverage to the molecular coverage

A full monolayer of surface Hf complexes corresponds to a density of 0.0115 Å^2, as was derived in section S5c. Using this number, the coverage in Fig. 2c can be translated into the actual surface Hf complex density, cf. Fig. 2d.

S6. Interpretation of the C 1s:N 1s intensity ratio

From the spectra in Fig. 1 we find an experimental C 1s:N 1s ratio of 1.5. In this section we determine whether this value agrees with the expected one based on the information on the surface-adsorbed complexes as derived in section S5. To this end, we adopt a method of intensity normalization similar to that used in section S5. In subsection S6a we analyze the gas phase C 1s:N 1s intensity ratio, which allows determination of all unknown experimental parameters and to summarize in a factor α value; in subsection S6b. we derive the expected C 1s:N 1s ratio and compare it the experimental one, finally, in section S6c, we provide details on the derivation of the the parameters used in subsections S6a and S6b.

S6a. TDMAHf gas phase C 1s: N 1s intensity ratio

Using the nomenclature of section S5a, the intensity ratio can be expressed as follows:

$$\frac{I_{C1s,g}}{I_{N1s,g}} =$$

$$= \frac{\left(n'_{3\,DMA^-} \cdot N_{C.3\,DMA^-} \cdot C_{C,3\,DMA^-} + n'_{4\,DMA^-} \cdot N_{C,4\,DMA^-} \cdot C_{C,4\,DMA^-} + n'_{DMA} \cdot N_{C,DMA} \cdot C_{C,DMA}\right) \cdot \alpha_{C1s}}{\left(n'_{3\,DMA^-} \cdot N_{N.3\,DMA^-} \cdot C_{N,3\,DMA^-} + n'_{4\,DMA^-} \cdot N_{N,4\,DMA^-} \cdot C_{N,4\,DMA^-} + n'_{DMA} \cdot N_{N,DMA} \cdot C_{N,DMA}\right) \cdot \alpha_{N1s}}. \quad (23)$$

Here, the fractions of molecules in the gas phase are expressed as n'; in contrast to the treatment in section S5, the entire intensities of the N 1s and the C 1s gas phase core levels spectra are used in the further procedure. We know from section S5, w that the DMA intensity accounts for 10 %

of the total N 1s intensity, and since $Hf(N(CH_3)_2)_3OH$ and DMA result from the same reaction in equal quantities, $n'_{3\,DMA^-} = n'_{DMA}$. As in section S5, we find a system of equations:

$$\begin{cases} n'_{4.DMA^-} + n'_{3.DMA^-} + n'_{DMA} = 1 \\ n'_{3.DMA^-} = n'_{DMA} \\ \dfrac{n'_{3\,DMA^-} \cdot N_{DMA} \cdot C_{DMA}}{(n'_{3\,DMA^-} \cdot N_{DMA} \cdot C_{DMA} + n'_{3\,DMA^-} \cdot N_{3\,DMA^-} \cdot C_{3\,DMA^-} + n'_{4\,DMA^-} \cdot N_{4\,DMA^-} \cdot C_{4\,DMA^-})} = 0.1, \end{cases} \quad (24)$$

We obtain $n'_{4.DMA^-} = 48.2\ \%$, $n'_{3.DMA^-} = n'_{DMA} = 25.9\ \%$.

With the coefficients just obtained and the fact that there are two carbon atoms per nitrogen atom irrespective of how many ligands are retained per complex, we find:

$$\frac{I_{C1s,g}}{I_{N1s,g}} = 2.186 \cdot \frac{\alpha_{C1s}}{\alpha_{N1s}}. \quad (25)$$

The experimental gas phase C 1s : N 1s ratio is 1.64; thus

$$\frac{\alpha_{C1s}}{\alpha_{N1s}} = 0.750. \quad (26)$$

S6b. C 1s: N 1s ratio in the complex adsorbed on the surface

Using the same notation as used in section S5 and S6a, we can write the expected ratio C 1s : N 1s as follows:

$$\frac{I_{C1s}}{I_{N1s}} = \frac{(n'_{2\,DMA^-} \cdot N_{C.2\,DMA^-} \cdot C_{C,2\,DMA^-} + n'_{4\,DMA^-} \cdot N_{C,4\,DMA^-} \cdot C_{C,4\,DMA^-})}{(n'_{2\,DMA^-} \cdot N_{N.2\,DMA^-} \cdot C_{N,2\,DMA^-} + n'_{4\,DMA^-} \cdot N_{N,4\,DMA^-} \cdot C_{N,4\,DMA^-})} \cdot \frac{\alpha_{C1s}}{\alpha_{N1s}} \quad (27)$$

$\frac{\alpha_{C1s}}{\alpha_{N1s}}$ is as in section S6a, $N_C = 2N_N$, and from section S5c.1 $n_{2\,DMA^-}$=74 %, $n_{4\,DMA^-}$= 26 %. The correction factors $C_{N,2\,DMA^-}$ and $C_{N,4\,DMA^-}$ are as in Table S1, and $C_{C,2\,DMA^-}$ and $C_{C,4\,DMA^-}$ are derived in section S6c. We find

$$\frac{I_{C1s}}{I_{N1s}} = 2.344 \cdot \frac{\alpha_{C1s}}{\alpha_{N1s}} = 2.344 \cdot 0.750 = 1.76. \tag{28}$$

This number is in excellent agreement with the experimental C 1s:N 1s intensity ratio of 1.5.

S6c. Internal attenuation of the C 1s signal from the gas phase and from the adsorbed complex

The internal attenuation of the C 1s XP signal in the gas phase as well as in the complex on the surface is estimated using the same approach as used in section S5, using an inelastic mean free path of 14 Å. The resulting factors are listed in Table S1. In deriving these factors, we assumed that the ligands of the surface-adsorbed complex with two retained DMA^- point outwards from the surface in a symmetric fashion. The coordinates of the carbon and hydrogen atoms relative to the Hf ion are assumed to be the same as in Ref. S45. In this way, we find that the two carbon atoms bond to each nitrogen have a distance of approx. d_1 =1.52 Å and d_1 =1.17 Å from surface of the sphere containing the complex. For the correction factor that describes the internal information of the surface-adsorbed complex with two retained ligands we find:

$$C_{C,2\,DMA^-} = \frac{1}{4} \cdot \left(2 \cdot e^{-\frac{d_1}{14}} + 2 \cdot e^{-\frac{d_2}{14}}\right) = 0.909. \tag{29}$$

S7. DFT results, decomposition of TDMAHf

S7a. Unimolecular gas-phase decomposition of TDMAHf

Decomposition of TDMAHf can proceed via four plausible mechanisms:[S55] *i*) formation of $CH_3N{=}CH_2$ molecule via β-hydride elimination, *ii*) formation of a DMA molecule via an intramolecular insertion reaction leading to metallacycle generation, *iii*) elimination of a methane

molecule via an intramolecular insertion reaction leading to a four-member ring and *iv*) formation of a methane molecule via a 1,2-β-hydride elimination reaction. It was found[S55] that, among these channels, the elimination of DMA has the lowest activation barrier (161.5 kJ/mol). This is consistent with the results of gas-phase FTIR analysis ,[S55] which indicated DMA as the main product of TDMAHf decomposition.

Here, we modeled only two decomposition reactions of isolated TDMAHf molecule (Figure S5). The first channel is the elimination of a DMA molecule. The second channel is the homolytic cleavage of the Hf-N bond. We find that the elimination of DMA from TDMAHf proceeds via two steps: The first step is the formation of a complex containing the DMA molecule and a Hf complex with a N-methyl methyleneimine (Hf-MMI) molecule residue. This step requires 178.1 kJ/mol. The reaction is endothermic at a calculated reaction energy of 115.7 kJ/mol. The second step is the fragmentation of the complex and elimination of DMA. This step requires an additional 152.5 kJ/mol. The overall activation energy required for elimination of DMA from TDMAHf in the gas phase requires 268.2 kJ/mol. In comparison, the homolytic cleavage of the Hf-N bond requires 381.0 kJ/mol.

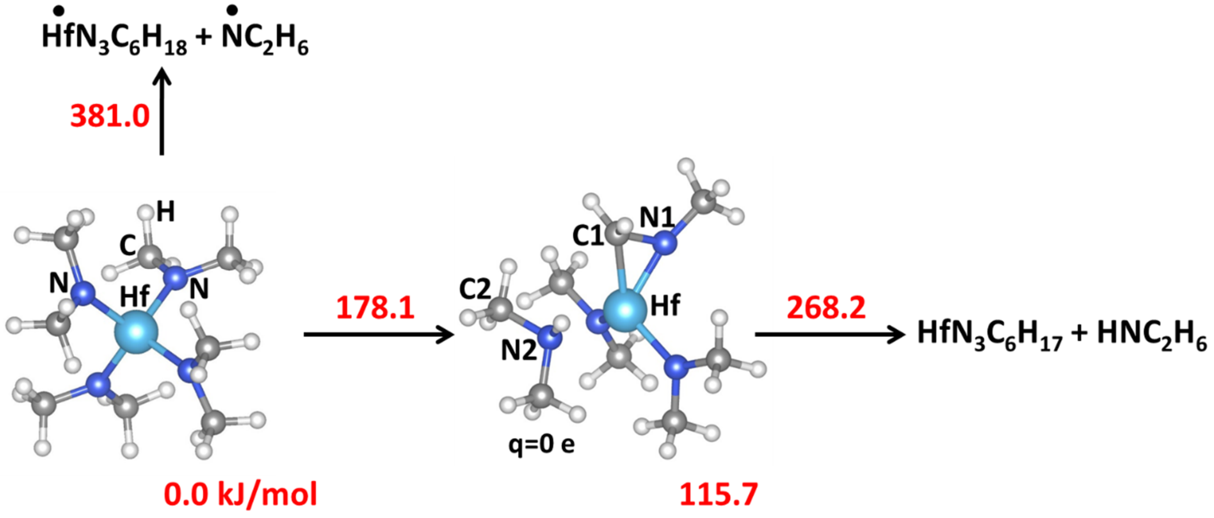

Figure S5. Decomposition of isolated TDMAHf molecule

S7b. Gas-phase decomposition in the TDMAHf dimer

The formation of a TDMAHf dimer is a possible outcome of the interaction of gas-phase TDMAHf molecules.[S55] While further decomposition of the dimer is usually not taken into account, here we model the elimination of DMA from a TDMAHf dimer. The rection energy diagram for this reaction is shown in Figure S6. The formation of the dimer is an energetically favorable process: TDMAHf dimer lies by 108.9 kJ/mol lower than two isolated molecules. The next reaction step (***C1***-***C2***) is the formation of a complex containing a MMI fragment and DMA. This step requires 165.2 kJ/mol, which is ~10 kJ/mol lower than what is required for the formation of DMA in an isolated TDMAHf. The reaction energy of this step is 73.8 kJ/mol. The final step of dimer decomposition is the elimination of DMA from the dimer complex (***C2***-***C3***). The results of our calculations show that the elimination of DMA requires 86.4 kJ/mol. Comparison of Figures S6 and S8 shows that the elimination of DMA from a TDMAHf dimer requires significantly lower energy (165.2 kJ/mol, Figure S6) than from isolated TDMAHf (268.2 kJ/mol, Figure S5).

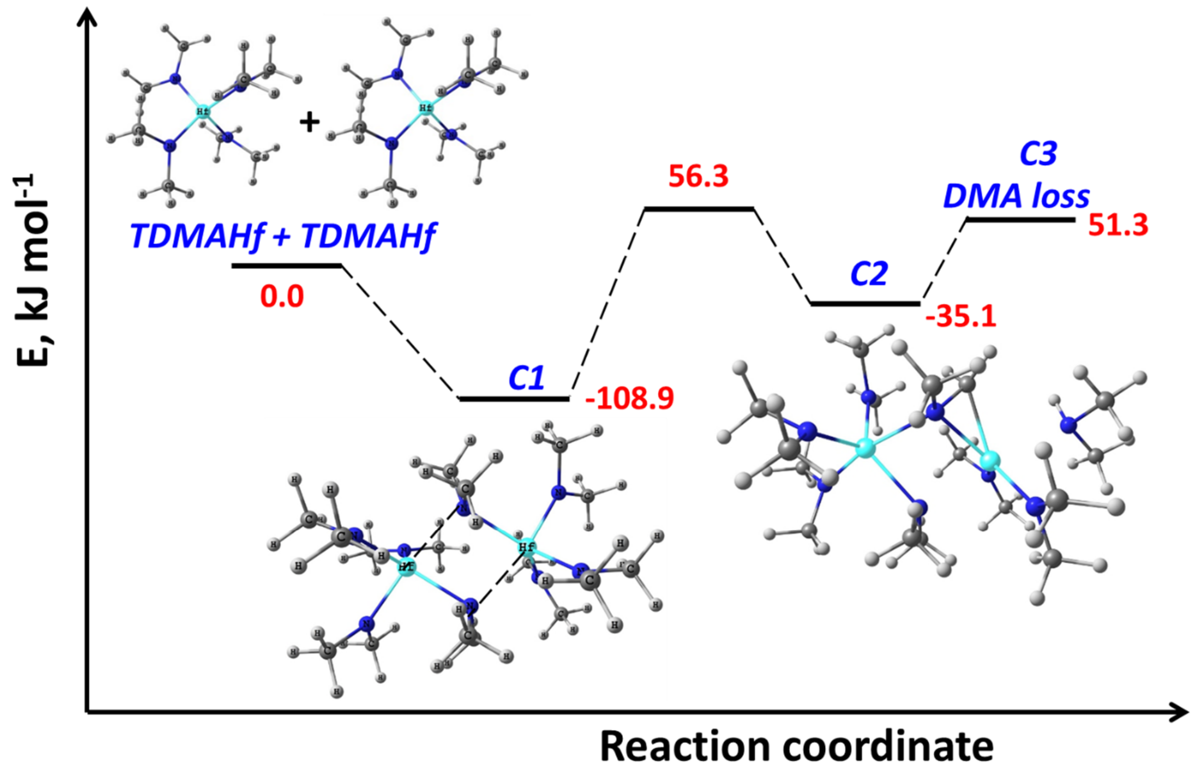

Figure S7. Decomposition of TDMAHf dimer via elimination of DMA molecule

S7c. Unimolecular decomposition of TDMAHf on a SiO_2(001) surface

To study the effect of the SiO_2 surface on the decomposition of TDMAHf, we modeled the interaction of TDMAHf with a pristine SiO_2(001) surface. TDMAHf physiosorbs on this surface with an adsorption energy of -76.7 kJ/mol (Figure S8). Further, our calculations show that the SiO_2(001) surface does not have any effect on the TDMAHf decomposition via DMA elimination and formation of a Hf complex with a MMI residue; the surface does not facilitate the decomposition reactions and the fragmentation of TDMAHf proceeds via the same mechanism as in the gas phase. In more detail, the first step (***D1-D2***) of TDMAHf decomposition on the SiO_2(001) surface is formation of a complex containing the MMI fragment and DMA. The second step (***D2-D3***) is elimination of DMA. The overall energy required for removal of DMA from the system is 306 kJ/mol.

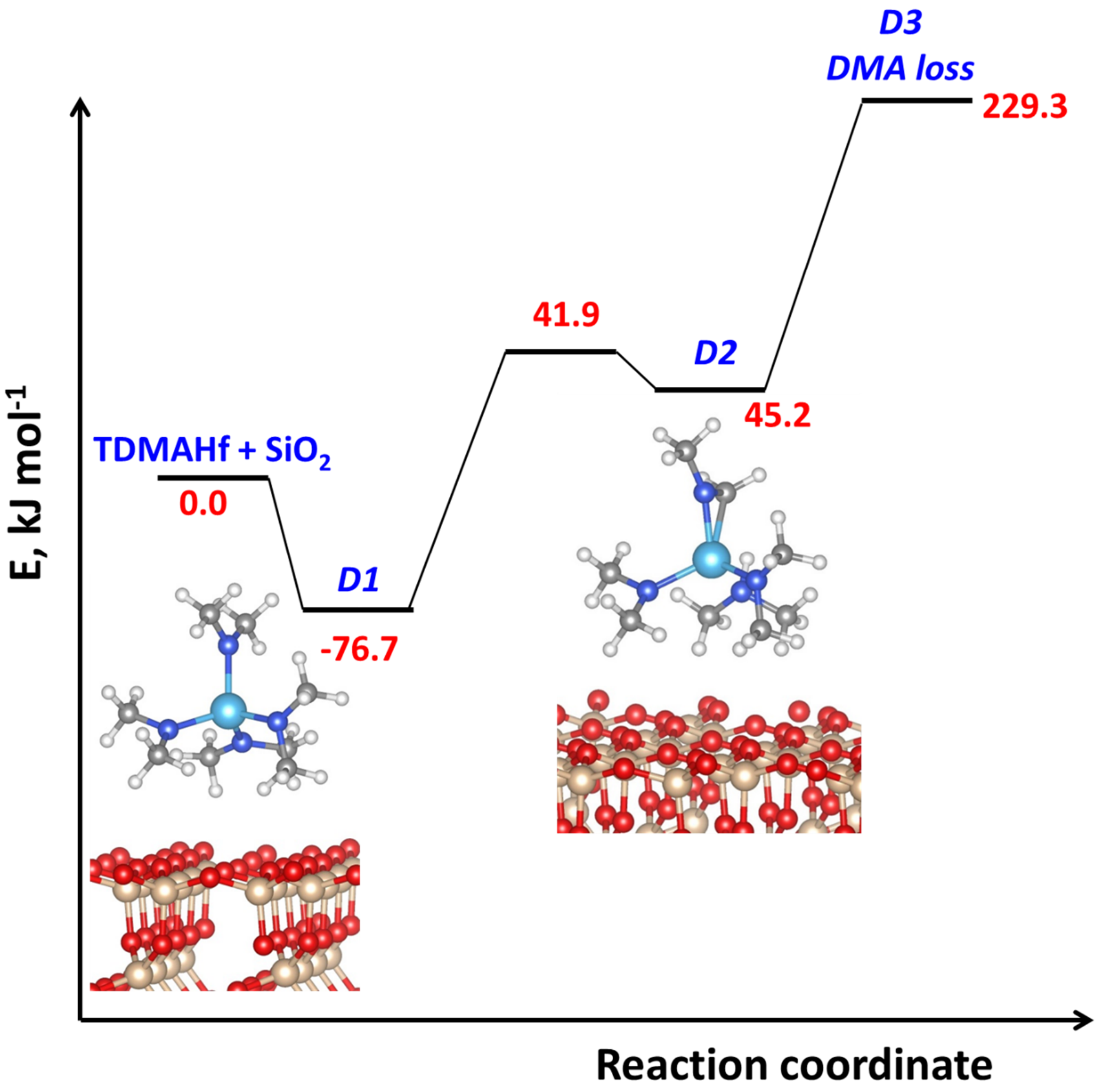

Figure S8. Decomposition of TDMAHf molecule on a pristine SiO_2(001) surface.

S7d. TDMAHf dimer formation on a SiO_2(001) surface and decomposition in the adsorbed dimer

The possible mechanisms of dimer formation and decomposition on the SiO_2(001) surface are shown in Figure S9. Formation of the TDMAHf dimer on the SiO_2(001) surface is a favorable process. The calculated formation energy is -160.4 kJ/mol. The subsequent decomposition of the

dimer towards a surface -Hf-MMA complex and DMA can proceed via two plausible mechanisms. The first mechanism (***E1-E2-E3***) involves intermolecular hydrogen transfer from a DMA^- ligand of one TDMAHf adsorbate to a DMA^- ligand of a neighboring TDMAHf molecule. The first step (***E1-E2***) is associated with formation of an MMI fragment via hydrogen transfer and requires 151 kJ/mol. The intermediate structure ***E2*** lies 99.7 kJ/mol above structure ***E1***. Subsequent removal of DMA (***E2-E3***) requires 13.3 kJ/mol. The overall rection energy of this first mechanism is -47.4 kJ/mol^{-1}. The second mechanism (***E1-E4-E5***) proceeds via intramolecular hydrogen transfer between DMA^- ligands of the same TDMAHf adsorbate within the TDMAHf dimer. The first step (***E1-E4***) corresponds to formation of a -Hf-MMI complex and DMA. This step has an activation barrier of 155.5 kJ/mol, whereas the intermediate structure ***E4*** lies 59.9 k/mol above ***E1***. Removal of DMA from the intermediate structure ***E4*** requires 70 kJ/mol. The overall rection energy of this second reaction mechanism is -30.5 kJ/mol.

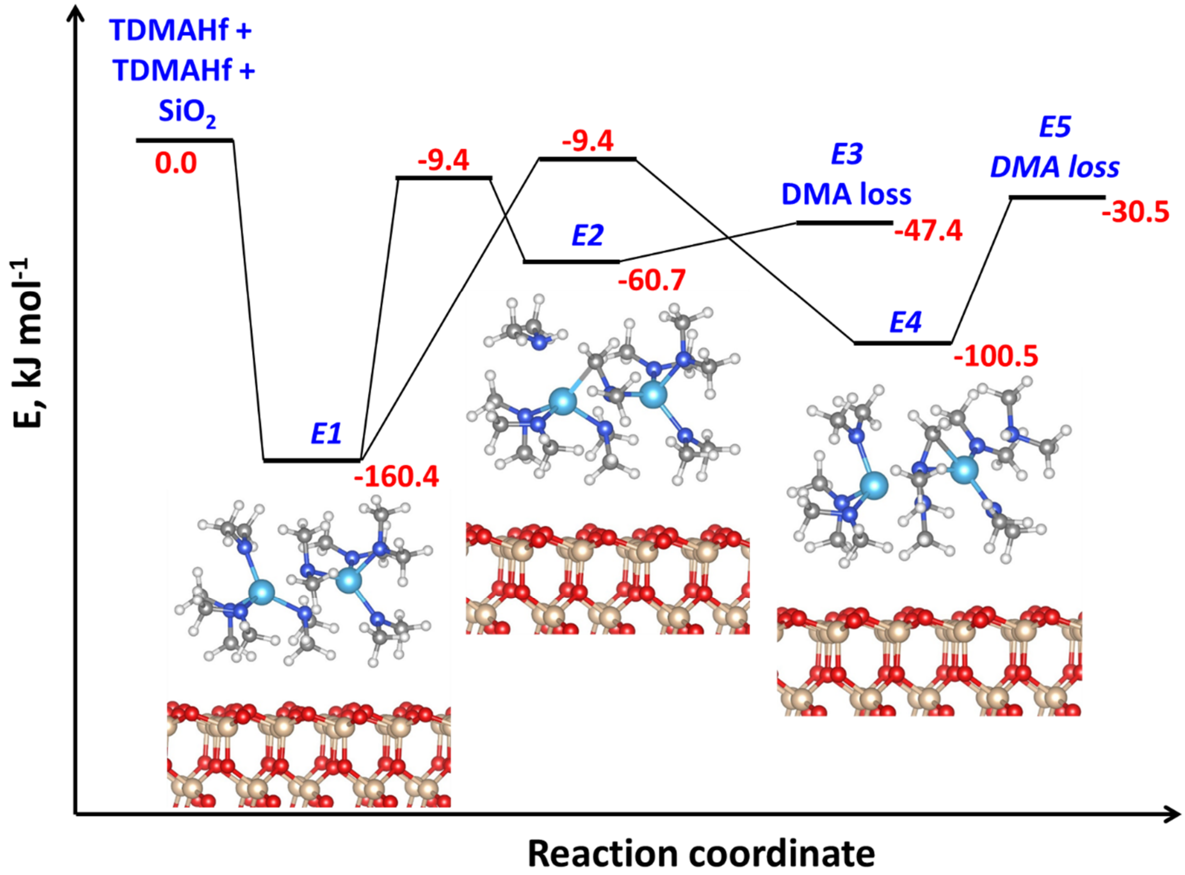

Figure S9. Decomposition of TDMAHf dimer on a pristine SiO_2(001) surface.

As already stated above, the results of our calculations show that the SiO_2(001) surface has little or no effect of the decomposition of TDMAHf. The energies calculated for TDMAHf decomposition on a pristine SiO_2(001) surface are very close to the energies of decomposition of isolated molecule (~306 kJ/mol vs 268 kJ/mol). Elimination of DMA from the system is the most demanding step energy wise and, hence, should be considered as rate-determining. Decomposition of the TDMAHf dimer requires significantly lower energies both in the gas phase (165 kJ/mol) and on the SiO_2(001) surface (151-155 kJ/mol) as compared to a unimolecular decomposition. In the dimer decomposition, the formation of the MMI fragment is the rate-limiting step, but not the removal of DMA. Furthermore, unlike the decomposition of a single TMDAHf molecule on the

SiO_2(001) surface, the decomposition of the dimer is a thermodynamically favorable process with an overall reaction energy -30.5 kJ/mol and -47.4 kJ/mol (Figure S9).

S8. Kinetic modelling

In order to evaluate whether the experimental temperature of 280 °C is sufficient to allow the different decomposition reaction mechanisms in Fig. 4 to proceed, we modelled their kinetics using the activation energy in the Figure. In Table S2, the activation energies E_a for all reactions, labelled as in Fig.4, and the corresponding rate constants are reported. The rate constants are estimated using the Arrhenius equation, with the pre-factor *A* assumed[S56] to be $1{,}00 \cdot 10^{13}\,\frac{1}{s}$:

$$k = A \cdot e^{-\frac{E_a}{k_B \cdot T}} \tag{30}$$

Reaction	Barrier	Designation of rate constant	Barrier [kJ/mol]	rate constant [1/s]
Unimolecular	A1-A2	k_2	150,4	6,28E-02
Unimolecular	A2-A1	k_3	79,3	3,25E+05
Unimolecular	A2-A3	k_4	192,2	7,09E-06
Bimolecular	B1-B2	k_2	172,6	5,03E-04
Bimolecular	B2-B1	k_3	80,8	2,34E+05
Bimolecular	B2-B3	k_4	22,3	7,84E+10
Bimolecular	B1-B4	k_2	153,7	3,06E-02
Bimolecular	B4-B1	k_3	17,0	2,48E+11
Bimolecular	B4-B5	k_4	20,6	1,13E+11

Table S2.Activation energies and rate constants for the three decomposition reaction mechanisms depicted in Fig. 4.

The kinetics of the three different reaction mechanisms are calculated by solving a set of coupled differential equations as follows. If we label the concentration of TDMAHf in the gas phase, the density of available (non-occupied) surface sites, the surface density of adsorbed TDMAHf, the

surface density of reacted TDMAHf (i.e. with an MMI ligand), the surface density of DMA adsorbates and the gas phase DMA concentration as [*A*], [*B*], [*C*], [*D*], [*E*] and [*F*], respectively, we can write, for the *unimolecular reaction* path with the corresponding species *A* to *F*:

$$A + B \overset{k_1}{\rightarrow} C \overset{k_3}{\leftarrow}\overset{k_2}{\rightarrow} D + E \overset{k_4}{\rightarrow} D + F$$

Here, k_1 is chosen as to reproduce the coverage evolution as shown in Fig. 2c. k_1 is proportional to the flux of impinging TDMAHf molecules, the sticking coefficient and the surface coverage. A value of k_1 of 0.03 s^{-1} mimics the experimental data well.

We can now write a set of six differential equations (one for each concentration):

$$\begin{cases} \frac{d[A]}{dt} = 0 \\ \frac{d[B]}{dt} = -k_1[A][B] \\ \frac{d[C]}{dt} = k_1[A][B] + k_3[D][E] - k_2[C] \\ \frac{d[D]}{dt} = -k_3[D][E] + k_2[C] \\ \frac{d[E]}{dt} = -k_2[D][E] + k_2[C] - k_4[E] \\ \frac{d[F]}{dt} = k_4[E] \end{cases}$$

This set of coupled differential equations is numerical solvable assuming as initial condition (at *t*=0) the following concentrations: [*A*(*t*=0)] and [*B*(*t*=0)] = 1, and all others concentrations 0.

The results of the numerically solved set of differential equations are shown in Fig. S10. Clearly, on the timescale of the experiment, there is no production of MMI or DMA (surface densities [*D*] and [*E*], respectively).

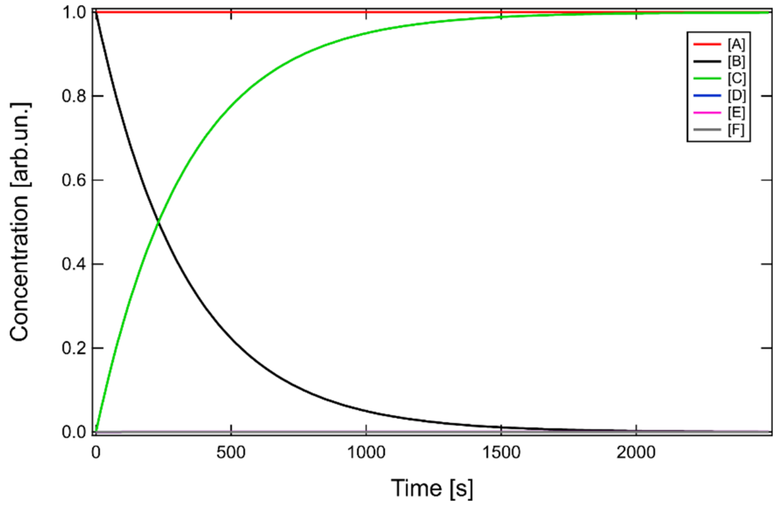

Figure S10. Result of the kinetic modelling of the unimolecular reaction path depicted in Fig. 4a.

Regarding the *bimolecular reactions*, the stoichiometric coefficient is adjusted to include the formation of a TDMAHf dimer with surface density $[D]$ from two adsorbed TDMAHf complexes with surface density $[C]$:

$$A + B \overset{k_1}{\rightarrow} C \overset{k_3}{\leftarrow}\overset{k_2}{\rightarrow} 0.5 \cdot D + 0.5 \cdot E \overset{k_4}{\rightarrow} 0.5 \cdot D + 0.5 \cdot F$$

This leads to the following set of coupled ODEs:

$$\begin{cases} \frac{d[A]}{dt} = 0 \\ \frac{d[B]}{dt} = -k_1[A][B] \\ \frac{d[C]}{dt} = k_1[A][B] + 2 \cdot k_3[D][E] - k_2[C]^2 \\ \frac{d[D]}{dt} = -k_3[D][E] + 0.5 \cdot k_2[C]^2 \\ \frac{d[E]}{dt} = -k_2[D][E] + 0.5 \cdot k_2[C]^2 - k_4[E] \\ \frac{d[F]}{dt} = k_4[E] \end{cases}$$

The numerical solution to this set of coupled differential equations is shown in Figs. S12 and S13, where the same initial conditions were assumed as in the monomolecular case above. Clearly, both

biomolecular pathways allow the formation of MMI (surface density [*D*], blue curve) and the release of DMA into the gas phase (concentration [*F*], gray curve) on the timescale of the experiment (note that the two curves are overlapping).

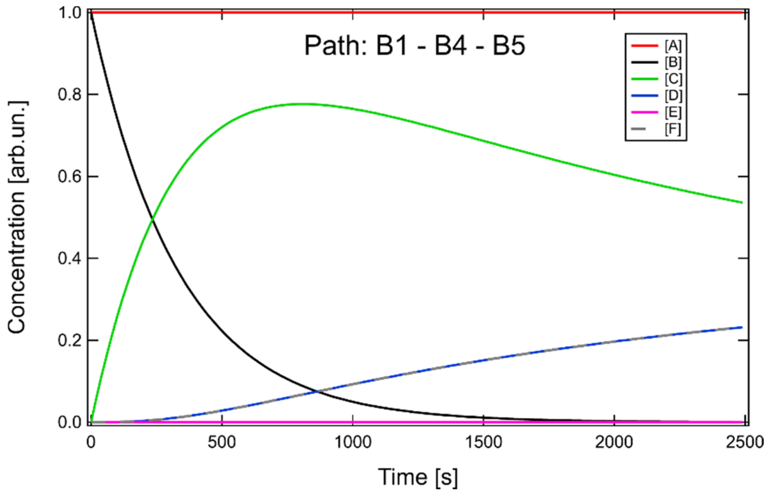

Figure S11. Result of the kinetic modelling of the bimolecular reaction path *B1-B2-B3* depicted in Fig. 4b.

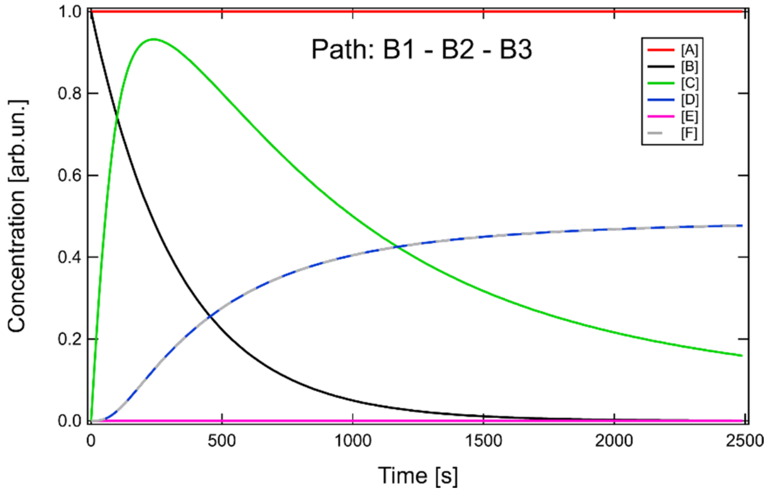

Figure S12. Result of the kinetic modelling of the bimolecular reaction path *B1-B4-B5* depicted in Fig. 4b.

References

S1. Gabriel, N. T.; Kim, S. S.; Talghader, J. J. Control of Thermal Deformation in Dielectric Mirrors Using Mechanical Design and Atomic Layer Deposition. *Opt. Lett.* **2009**, *34*, 1958. https://doi.org/10.1364/OL.34.001958

S2. Gabriel, N. T.; Talghader, J. J. Optical Coatings in Microscale Channels by Atomic Layer Deposition. *Opt. Lett.* **2010**, *49*, 1242. https://doi.org/10.1364/AO.49.001242

S3. Moriyama, N.; Ohno, Y.; Kitamura, T.; Kishimoto, S.; Mizutani, T. Change in Carrier Type in High-k Gate Carbon Nanotube Field-Effect Transistors by Interface Fixed Charges. *Nanotechnology* **2010**, *21*, 165201. https://doi.org/10.1088/0957-4484/21/16/165201

S4. Kim, J. C.; Cho, Y. S.; Moon, S. H. Atomic Layer Deposition of HfO_2 onto Si Using $Hf(NMe_2)_4$. *Jpn. J. Appl. Phys.* **2009**, *48*, 066515 https://doi.org/10.1143/JJAP.48.066515

S5. Platt, C. L.; Li, N.; Li, K.; Klein, T.M. Atomic Layer Deposition of HfO_2: Growth Initiation Study on Metallic Underlayers. *Thin Solid Films* **2010**, *518*, 4081–4086. https://doi.org/10.1016/j.tsf.2009.10.146

S6. Kelly, M. J.; Han, J. H.; Musgrave, C. B.; Parsons, G. N. *In-Situ* Infrared Spectroscopy and Density Functional Theory Modeling of Hafnium Alkylamine Adsorption on Si-OH and Si-H Surfaces. *Chem. Mater.* **2005**, *17*, 5305-5314. https://doi.org/10.1021/cm051064h

S7. Li, K.; Li, N.; Li, S.; Dixon, D. A.; Klein, T. M. Tetrakis(dimethylamido) Hafnium Adsorption and Reaction on Hydrogen-Terminated Si(100) Surfaces. *J. Phys. Chem. C* **2010**, *114*, 14061–14075. https://doi.org/10.1021/jp101363r

S8. Oh, I.L.; Park, B.-E.; Seo, S.; Yeo, B. C.; Tanskanen, J.; Lee, H. B. R.; Kim, W. H.; Kim, H. Comparative Study of the Growth Characteristics and Electrical Properties of Atomic-Layer Deposited HfO_2 Films Obtained from Metal Halide and Amide Precursors. *J. Mater. Chem. C* **2018**, *6*, 7367. https://doi.org/10.1039/c8tc01476k

S9. Hausmann, D. M.; Kim, E.; Becker, J.; Gordon, R. G. Atomic Layer Deposition of Hafnium and Zirconium Oxides Using Metal Amide Precursors. *Chem. Mater.* **2002**, 14, 4350-4358. https://doi.org/10.1021/cm020357x

S10. Hausmann, D. M.; Gordon, R. G. Surface Morphology and Crystallinity Control in the Atomic Layer Deposition (ALD) of Hafnium and Zirconium Oxide Thin Films. *J. Crystal Growth* **2003**, *249*, 251. https://doi.org/10.1016/S0022-0248(02)02133-4

S11. Kukli, K.; Pilvi, T.; Ritala, M.; Sajavaara, T.; Lu, J.; Leskelä, M. Atomic Layer Deposition of Hafnium Dioxide Thin Films from Hafnium Tetrakis(dimethylamide) and Water. *Thin Solid Films* **2005**, *491*, 328 – 338. https://doi.org/10.1016/j.tsf.2005.05.050

S12. Hong, J.; Porter, D. W.; Sreenivasan, R.; McIntyre, P. C.; Bent S. F. ALD Resist Formed by Vapor-Deposited Self-Assembled Monolayers. *Langmuir* **2007**, *23*, 1160-1165. https://doi.org/10.1021/la0606401

S13. Hackley, J. C.; Demaree, J. D.; Gougousi, T. Growth and Interface of HfO_2 Films on H-Terminated Si from a TDMAH and H_2O Atomic Layer Deposition Process. *J. Vac. Sci. Technol. A* **2008**, *26*, 1235. https://doi.org/10.1116/1.2965813

S14. O'Mahony, A.; Pemble, M. E.; Povey I. M. Infrared and Near-infrared spectroscopic probing of atomic layer deposition processes. *J. Mol. Struct.* **2010**, *976*, 324–327. https://doi.org/10.1016/j.molstruc.2010.03.087

S15. Kolanek, K.; Tallarida, M.; Karavaev K.; Schmeisser, D. *In Situ* Studies of the Atomic Layer Deposition of Thin HfO_2 Dielectrics by Ultra High Vacuum Atomic Force Microscope. *Thin Solid Films* **2010**, *518*, 4688–4691. https://doi.org/10.1016/j.tsf.2009.12.060

S16. Maeng, W. J.; Kimzet, H. Electrical Properties of Atomic Layer Deposition HfO_2 and HfO_xN_y on Si Substrates with Various Crystal Orientations. *J. Electrochem. Soc.* **2008**, *155*, 267. https://doi.org/10.1149/1.2840616

S17. Blaschke, D.; Munnik, F.; Grenzer, J.; Rebohle, L.; Schmidt, H.; Zahn, P.; Gemming, S. A Correlation Study of Layer Growth Rate, Thickness Uniformity, Stoichiometry, and Hydrogen Impurity Level in HfO_2 Thin Films Grown by ALD between 100 °C and 350 °C. *Appl. Surf. Sci.* **2020**, *506*, 144188. https://doi.org/10.1016/j.apsusc.2019.144188

S18. Ko, B. G.; Nguyen, C. T.; Gu, B.; Khan, M. R.; Park, K.; Oh, H.; Park, J.; Shong, B.; Lee, H.-B.-R. Growth Modulation of Atomic Layer Deposition of HfO_2 by Combinations of H_2O and O_3 Reactants. *Dalton Trans.*, **2021**, *50*, 17935. https://doi.org/10.1039/d1dt03465k

S19. Oh, I.-K.; Tanskanen, J.; Jung, H.; Kim, K.; Lee, M. J.; Lee, Z.; Lee, S.-K.; Ahn, J.-H.; Lee, C. W.; Kim, K.; Kim, H.; Lee, H.-B.-R. Nucleation and Growth of the HfO_2 Dielectric Layer for Graphene-Based Devices. *Chem. Mater.* **2015**, *27*, 5868−5877. https://doi.org/10.1021/acs.chemmater.5b01226

S20. Guo, L.; Zaera, F. Spatial Resolution in Thin Film Deposition on Silicon Surfaces by Combining Silylation and UV/Ozonolysis. *Nanotechnology* **2014**, *25*, 504006. https://doi.org/10.1088/0957-4484/25/50/504006

S21. Oh, I.-K.; Kim, M.-K.; Lee, J.-S.; Lee, C.-W.; Lansalot-Matras, C.; Noh, W.; Park, J.; Noori, A.; Thompson, D.; Chud, S.; Maenge, W. J.; Kim, H. The Effect of La_2O_3-Incorporation in HfO_2 Dielectrics on Ge Substrate by Atomic Layer Deposition. *Appl. Surf. Sci.* **2013**, *287*, 349– 354. https://doi.org/10.1016/j.apsusc.2013.09.153

S22. Maeng, W. J.; Oh, I.-K.; Kim, W.-H.; Kim, M.-K., Lee, C.-W.; Lansalot-Matras, C.; Thompson, D.; Chue, S.; Kim, H. Atomic Layer Deposition of CeO_2/HfO_2 Gate Dielectrics on Ge Substrate. *Appl. Surf. Sci.* **2014**, *321*, 214–218. https://doi.org/10.1016/j.apsusc.2014.10.025

S23. Abdel-Fattah, T. M.; Gu, D.; Baumgart, H. Atomic Layer Deposition Grown Hafnia Nanotubes Functionalized with Gold Nanoparticle Composites. *ECS Solid State Lett.* **2013**, *2*, P31-P34. https://doi.org/10.1149/2.001303ssl

S24. Hohenberg, P.; Kohn, W. Inhomogeneous Electron Gas. *Phys. Rev*. **1964**, *136*, B864. https://doi.org/10.1103/PhysRev.136.B864

S25. Zhao, Y.; Truhlar, D. G. The M06 Suite of Density Functionals for Main Group Thermochemistry, Thermochemical Kinetics, Noncovalent Interactions, Excited States, and Transition Elements: Two New Functionals and Systematic Testing of Four M06-Class Functionals and 12 Other Functionals. *Theor. Chem. Acc*. **2008**, *120*, 215. https://doi.org/10.1007/s00214-007-0310-x

S26. Frisch, M. J.; Trucks, G. W.; Schlegel, H. B.; Scuseria, G. E.; Robb, M. A.; Cheeseman, J. R.; Scalmani, G.; Barone, V.; Petersson, G. A.; Nakatsuji, H.; Li, X.; Caricato, M.; Marenich, A. V.; Bloino, J.; Janesko, B. G.; Gomperts, R.; Mennucci, B.; Hratchian, H. P.; Ortiz, J. V.; Izmaylov, A. F.; Sonnenberg, J. L.; Williams; Ding, F.; Lipparini, F.; Egidi, F.; Goings, J.; Peng, B.; Petrone, A.; Henderson, T.; Ranasinghe, D.; Zakrzewski, V. G.; Gao, J.; Rega, N.; Zheng, G.; Liang, W.; Hada, M.; Ehara, M.; Toyota, K.; Fukuda, R.; Hasegawa, J.; Ishida, M.; Nakajima, T.; Honda, Y.; Kitao, O.; Nakai, H.; Vreven, T.; Throssell, K.; Montgomery Jr., J. A.; Peralta, J. E.; Ogliaro, F.; Bearpark, M. J.; Heyd, J. J.; Brothers, E. N.; Kudin, K. N.; Staroverov, V. N.; Keith, T. A.; Kobayashi, R.; Normand, J.; Raghavachari, K.; Rendell, A. P.; Burant, J. C.; Iyengar, S. S.; Tomasi, J.; Cossi, M.; Millam, J. M.; Klene, M.; Adamo, C.; Cammi, R.; Ochterski, J. W.; Martin, R. L.; Morokuma, K.; Farkas, O.; Foresman, J. B.; Fox, D. J. Gaussian 16 Rev. B.01; *Wallingford*, CT, **2016**.

S27. Hay, P. J.; Wadt, W. R. Ab Initio Effective Core Potentials for Molecular Calculations. Potentials for the Transition Metal Atoms Sc to Hg. *J. Chem. Phys.* **1985**, *82*, 270. https://doi.org/10.1063/1.448799

S28. Hay, P. J.; Wadt, W. R. Ab Initio Effective Core Potentials for Molecular Calculations. Potentials for K to Au Including the Outermost Core Orbitals. *J. Chem. Phys.* **1985**, *82*, 299. https://doi.org/10.1063/1.448975

S29. Wadt, W. R.; Hay, P. J. Ab Initio Effective Core Potentials for Molecular Calculations. Potentials for Main Group Elements Na to Bi. *J. Chem. Phys.* **1985**, *82*, 284. https://doi.org/10.1063/1.448800

S30. Dion, M.; Rydberg, H.; Schröder, E.; Langreth, D. C.; Lundqvist, B. I. Van Der Waals Density Functional for General Geometries. *Phys. Rev. Lett*. **2004**, *92*, 246401. https://doi.org/10.1103/PhysRevLett.92.246401

S31. Thonhauser, T.; Cooper, V. R.; Li, S.; Puzder, A.; Hyldgaard, P.; Langreth, D. C. Van Der Waals Density Functional: Self-Consistent Potential and the Nature of the van Der Waals Bond. *Phys. Rev. B* **2007**, *76*, 125112. https://doi.org/10.1103/PhysRevB.76.125112

S32. Román-Pérez, G.; Soler, J. M. Efficient Implementation of a van Der Waals Density Functional: Application to Double-Wall Carbon Nanotubes. *Phys. Rev. Lett*. **2009**, *103*, 096102. https://doi.org/10.1103/PhysRevLett.103.096102

S33. Klimeš, J.; Bowler, D. R.; Michaelides, A. Chemical Accuracy for the van Der Waals Density Functional. *J. Phys.: Condens. Matter* **2010**, *22*, 022201. https://doi.org/10.1088/0953-8984/22/2/022201

S34. Klimeš, J.; Bowler, D. R.; Michaelides, A. Van Der Waals Density Functionals Applied to Solids. *Phys. Rev. B* **2011**, *83*, 195131. https://doi.org/10.1103/PhysRevB.83.195131

S35. Kresse, G.; Furthmüller, J. Efficiency of Ab-Initio Total Energy Calculations for Metals and Semiconductors Using a Plane-Wave Basis Set. *Comp. Mater. Sci.* **1996**, *6*, 15. https://doi.org/10.1016/0927-0256(96)00008-0

S36. Kresse, G.; Furthmüller, J. Efficient Iterative Schemes for Ab Initio Total-Energy Calculations Using a Plane-Wave Basis Set. *Phys. Rev. B* **1996**, *54*, 11169. https://doi.org/10.1103/PhysRevB.54.11169

S37. Kresse, G.; Hafner, J. Ab Initio Molecular Dynamics for Liquid Metals. *Phys. Rev. B* **1993**, *47*, 558. https://doi.org/10.1103/PhysRevB.47.558

S38. Blöchl, P. E. Projector Augmented-Wave Method. *Phys. Rev. B* **1994**, *50*, 17953. https://doi.org/10.1103/PhysRevB.50.17953

S39. Hom, T.; Kiszenik, W.; Post, B. Accurate Lattice Constants from Multiple Reflection Measurements. II. Lattice Constants of Germanium Silicon, and Diamond. *J. Appl. Crystallogr.* **1975**, *8*, 457. https://doi.org/10.1107/S0021889875010965

S40. Tao, F.; Xu, G. Q. Attachment Chemistry of Organic Molecules on Si(111)-7 × 7. *Acc. Chem. Res.* **2004**, *37*, 882. https://doi.org/10.1021/ar0400488

S41. Lee, S.-H.; Kang, M.-H. Origin of O 1s Core-Level Shifts on Oxygen Adsorbed Si(111)-(7×7). *Phys. Rev. Lett.* **2000**, *84*, 1724. https://doi.org/10.1103/PhysRevLett.84.1724

S42. Antao, S. M.; Hassan, I.; Wang, J.; Lee, P. L.; Toby, B. H. State-of-the-Art High-Resolution Powder X-ray Diffraction (HRPXRD) Illustrated with Rietveld Structure Refinement of Quartz, Sodalite, Tremolite, and Meionite. *Can. Mineral.* **2008**, *46*, 1501. https://doi.org/10.3749/canmin.46.5.1501

S43. Goumans, T. P. M.; Wander, A.; Brown, W. A.; Catlow, C. R. A. Structure and Stability of the (001) α-Quartz Surface. *Phys. Chem. Chem. Phys.* **2007**, *9*, 2146. https://doi.org/10.1039/B701176H

S44. Henkelman, G.; Uberuaga, B. P.; Jónsson, H. A Climbing Image Nudged Elastic Band Method for Finding Saddle Points and Minimum Energy Paths. *J. Chem. Phys.* **2000**, 113, 9901. https://doi.org/10.1063/1.1329672

S45. Shayesteh, P.; Tsyshevsky, R.; Urpelainen, S.; Rochet, F.; Bournel, F.; Gallet, J.-J.; Kuklja, M. M.; Schnadt, J.; Head, A. R. Experimental and Theoretical Gas Phase Electronic Structure Study of Tetrakis(Dimethylamino) Complexes of Ti(IV) and Hf(IV). *J. Electron Spectrosc. Relat. Phenom*. **2019**, *234*, 80. https://doi.org/10.1016/j.elspec.2019.05.016

S46. Kim, H.-L.; Kim, H.; Byun, Y. Lee, J.; Lee, W.-J. Surface Reaction of the Hafnium Precursors with a Linked Amido-Cyclopentadienyl Ligand: A Density Functional Theory Study. *J. Vac. Sci. Technol. A* **2021**, *39*, 032410. https://doi.org/10.1116/6.0000796

S47. Farfan-Arribas, E.; Madix, R. J. Characterization of the Acid−Base Properties of the TiO_2(110) Surface by Adsorption of Amines. *J. Phys. Chem. B* **2003**, *107*, 3225. https://doi.org/10.1021/jp022344c

S48. Almbladh, C.-O.; Hedin, L. Beyond the one-electron model: Many-body effects in atoms, molecules, and solid. In: *Handbook on Synchrotron Radiation*; Koch, E.-E., Ed.; vol. 1B; North-Holland: Amsterdam, 1983; vol 1B, pp 607-904.

S49. Tanuma, S.; Powell, C. J.; Penn, D. R. Calculations of Electron Inelastic Mean Free Paths. V. Data for 14 Organic Compounds over the 50-2000 eV Range. *Surf. Interface Anal.* **1994**, *21*, 165. https://doi.org/10.1002/sia.740210302

S50. Wang, S.; He, J.; Zhang, Y.; Xu, G. Q. Adsorption of O_2 and CO_2 on the Si(111)-7×7 Surfaces. *Surf. Sci*. **2012**, *606*, 1387. https://doi.org/10.1016/j.susc.2012.04.026

S51. Sakamoto, K.; Zhang, H. M.; Uhrberg, R. I. G. Observation of Two Metastable Oxygen Species Adsorbed on a Si (111)-(7×7) Surface: Reinterpretation of the Initial Oxidation Process. *Phys. Rev. B* **2003**, *68*, 075302. https://doi.org/10.1103/PhysRevB.68.075302

S52. Gallet, J.-J.; Silly, M. G.; Kazzi, M. E.; Bournel, F.; Sirotti, F.; Rochet, F. Chemical and Kinetic Insights into the Thermal Decomposition of an Oxide Layer on Si(111) from Millisecond Photoelectron Spectroscopy. *Sci. Rep.* **2017**, *7*, 14257. https://doi.org/10.1038/s41598-017-14532-4

S53. Post, P.; Wurlitzer, L.; Maus-Friedrichs, W.; Weber, A. Characterization and Applications of Nanoparticles Modified In-Flight with Silica or Silica-Organic Coatings. *Nanomaterials* **2018**, *8*, 530. https://doi.org/10.3390/nano8070530

S54. Ma, J. W.; Lee, W. J.; Bae, J. M.; Jeong, K. S.; Oh, S. H.; Kim, J. H.; Kim, S.-H.; Seo, J.-H.; Ahn, J.-P.; Kim, H.; Cho, M.-H. Carrier Mobility Enhancement of Tensile Strained Si and SiGe Nanowires via Surface Defect Engineering. *Nano Lett*. **2015**, *15*, 7204. https://doi.org/10.1021/acs.nanolett.5b01634

S55. Li, K.; Li, S.; Li, N.; Dixon, D. A.; Klein, T. M. Tetrakis(Dimethylamido)Hafnium Adsorption and Reaction on Hydrogen Terminated Si(100) Surfaces. *J. Phys. Chem. C* **2010**, *114*, 14061. https://doi.org/10.1021/jp101363r.

S56. Khawam, A.; Flanagan, D. R. Solid-State Kinetic Models: Basics and Mathematical Fundamentals. *J. Phys. Chem. B* **2006**, *110*, 17315. https://doi.org/10.1021/jp062746a
